# Asymptotics Near Extinction for Nonlinear Fast Diffusion on a Bounded Domain

**DOI:** 10.1007/s00205-023-01850-3

**Published:** 2023-02-25

**Authors:** Beomjun Choi, Robert J. McCann, Christian Seis

**Affiliations:** 1 Department of Mathematics, POSTECH, Pohang, Gyeongbuk South Korea; 2 Department of Mathematics, University of Toronto, Toronto, ON Canada; 3 Institut für Analysis und Numerik, Westfälische Wilhelms-Universität Münster, Münster, Germany

## Abstract

On a smooth bounded Euclidean domain, Sobolev-subcritical fast diffusion with vanishing boundary trace is known to lead to finite-time extinction, with a vanishing profile selected by the initial datum. In rescaled variables, we quantify the rate of convergence to this profile uniformly in relative error, showing the rate is either exponentially fast (with a rate constant predicted by the spectral gap), or algebraically slow (which is only possible in the presence of non-integrable zero modes). In the first case, the nonlinear dynamics are well-approximated by exponentially decaying eigenmodes up to at least twice the gap; this refines and confirms a 1980 conjecture of Berryman and Holland. We also improve on a result of Bonforte and Figalli by providing a new and simpler approach which is able to accommodate the presence of zero modes, such as those that occur when the vanishing profile fails to be isolated (and possibly belongs to a continuum of such profiles).

## Introduction

Setting $m_q:= 1 -\frac{2}{n+q}$ [26], consider the fast diffusion equation with the exponent $0<m \in ({m_{1-\frac{n}{2}}},1)$, integrable non-negative initial data, and Dirichlet boundary conditions, on a smooth bounded domain $\Omega \subset {\textbf{R}}^n$:

1.1 $$\begin{aligned} \begin{aligned} w_\tau = \Delta (w^m) \text { on } \Omega \\ w=0 \text { on } \partial \Omega . \end{aligned}\end{aligned}$$

This equation models heat flow in a material whose thermal conductivity $m w^{m-1}$ depends inversely on its local temperature *w*. With $m=1/2$ this has also been used to model the diffusion of plasma ions across a magnetic field in simulations [43] and experiments [51]. For such an initial value problem, it is known that the vanishing Dirichlet boundary conditions drive the solution *w* to become extinct in finite time, e.g., [15, 45, 46]. To understand the vanishing profile, rescale around the extinction time $T>0$,

$$\begin{aligned} \begin{aligned} { w(x,\tau )= \left( (1-m)(T-\tau )\right) ^{\frac{1}{1-m}} v^{\frac{1}{m}}(x,t), \quad t= \frac{m}{1-m} \ln \frac{T}{T-\tau }}, \end{aligned}\end{aligned}$$

to obtain an equation for *v*(*x*, *t*) with $p=1/m$:

1.2 $$\begin{aligned} \begin{aligned} \frac{\partial \,}{\partial t} \left( \frac{v^p}{p}\right) = \Delta v + v^p \text { on } \Omega \\ v=0 \text { on }\partial \Omega . \end{aligned}\end{aligned}$$

The rescaled solution *v* on $\Omega$ is known to converge (both subsequentially [15] and sequentially [32]) as $t\rightarrow \infty$ to some unique profile *V*(*x*) selected by the initial datum; moreover, *V* is a positive solution to the stationary elliptic problem

1.3 $$\begin{aligned} \begin{aligned} \Delta V + V^p=0 \text { on } \Omega \\ V=0 \text { on } \partial \Omega . \end{aligned}\end{aligned}$$

Solutions to (1.3) represent critical points of the Lyapunov functional [14]

1.4 $$\begin{aligned} E(v)=\frac{1}{2}\Vert \nabla v\Vert ^{ 2}_{L^2(\Omega )} -\frac{1}{p+1} \Vert v\Vert _{L^{p+1}(\Omega )}^{p+1} \end{aligned}$$

for the dynamics (1.2). Sobolev subcriticality $p^{-1} > {m_{1-\frac{n}{2}}} =\frac{n-2}{n+2}$ implies $L^{p+1}$-coercivity of the energy on the $L^{p+1}$-unit sphere, from which the existence of positive steady states (1.3) had earlier been derived by Berger [10]. Brezis and Nirenberg showed such solutions need not be unique however [16]; other uniqueness and nonuniqueness results concerning positive solutions in specific domain geometries may be found in the works of Gidas–Ni–Nirenberg [33] on the ball, Dancer [23, 24] on connected approximations to disjoint unions of balls, Damascelli–Grossi–Pacella [25] on domains with symmetry, Zou [52] on rough balls, Akagi–Kajikiya [4] who used instability to show that uniqueness fails on thin annuli, so solutions and their rotations form continuous families, and Akagi [5] who showed the stability of energy minimizers. On the other hand, Feireisl–Simondon [32] showed that the evolution (1.2) selects and converges to one of the positive solutions *V* of (1.3) (which may depend on the initial datum) as $t\rightarrow \infty$. They did not give a result on the convergence rate. Later Bonforte et al. [12] showed convergence in relative error $h:=\frac{v-V}{V}$, i.e.

1.5 $$\begin{aligned} \begin{aligned} \lim _{t\rightarrow \infty } \left\| \frac{v(x,t)}{V(x)} -1 \right\| _{L^\infty (\Omega )} = 0, \end{aligned}\end{aligned}$$

and provided an exponential rate of convergence in entropy sense, under a non-degeneracy condition which they were able to verify for *m* close to 1.

It has been a problem of considerable interest (a) to quantify the rate of convergence unconditionally, and (b) to predict the higher-order asymptotics of the relative error *h*. In contrast to the analogous questions set on the full space $\Omega = {\textbf{R}}^n$, resolved in [26] and its references [8, 21, 22, 29, 39, 44], this challenge is compounded by the fact that the linearized problem has unstable modes (including those corresponding to $\tau$-translations in the original variables, which blow up at different times *T*), and can also have zero modes, including modes called *integrable* that arise e.g. for reasons of symmetry, as for the thin annuli mentioned above. Recently, Bonforte and Figalli overcame some of these challenges to solve (a) for $C^{2,\alpha }$-generic smooth domains including the ball [11]. In a suitable Hilbert space, they show the linearized evolution of *h* is generated by a self-adjoint operator possessing a complete basis of eigenfunctions. To summarize their findings: the unstable (negative) modes cannot be active due to Feireisl and Simondon’s convergence, and neutral (zero) modes are absent on generic domains [50], in which case they show that the relative error decays uniformly with an exponential rate $\lambda$ no smaller than the first positive eigenvalue. On arbitrary smooth domains however, such a result eludes their techniques, which rely on the kernel of the linearized operator being trivial, and hence the limiting profile being isolated. A simpler derivation of the rate of exponential convergence was subsequently obtained under the same restriction by Akagi [6]; although he expresses convergence in terms of an energy rather than the entropy or relative error, these quantities can be compared using the boundary regularity theory of [37]. Finally, Jin and Xiong showed unconditionally that the rate of convergence is at least algebraic in *t* [36], but with a power that is not explicit. In the present manuscript, we bridge this wide gap (between exponential upper and algebraic lower bounds on the rate) by developing a new approach which yields that $\Vert h\Vert _{L^\infty }$ either decays exponentially with rate $\lambda$ or else decays algebraically at a rate 1/*t* or slower. In the second case, not only must zero modes be present, but they must be non-integrable in a sense made precise below. Moreover, when the decay rate is exponential, we address (b) by showing that the longtime asymptotics of the nonlinear problem are described by the linearized dynamics up to the error $e^{-2\lambda t}$ produced by quadratic corrections. This refines and confirms a conjecture made for the case $n=1$ by Berryman and Holland [15].

Besides being more powerful, our approach is also simpler than Bonforte and Figalli’s. Instead of augmenting Del Pino and Dolbeault’s nonlinear entropy method [29] with approximate orthogonality conditions, we rely on an ODE lemma of Merle and Zaag [42], which implies that the dynamics are eventually dominated either by stable or neutral modes. We must also control the nonlinearity by adapting parabolic regularity estimates to the geometry of the steady state around the domain boundary. Delicately matching these estimates to the ODE argument allows us to estimate the rate of convergence via the dichotomy described above. From there we use Hilbert projection techniques to get an asymptotic expansion up to an $$e^{-2\lambda t}$$ error. The latter resembles Denzler, Koch and McCann’s treatment of higher-order asymptotics in the narrower range $$m_0=1-\frac{2}{n}<m<1$$ of evolutions on the unbounded domain $$\Omega ={\textbf{R}}^n$$—though the linearized analysis around the selfsimilar spreading solution described in [26, 27] and their references is more subtle than the present problem, and our rate estimate does not require us to establish differentiable dependence of the flow on initial conditions. On the other hand, the whole space problem is not plagued by the multiplicity and continua of limiting profiles that we presently face. In the current setting, finer aspects of the dynamics (beyond rate $$2\lambda $$) may conceivably be described by constructing invariant manifolds, but we defer the exploration of this possibility to future research. In the porous medium regime (which refers to the complementary range of nonlinearities $$m>1)$$ on the whole space $$\Omega ={\textbf{R}}^n$$, such a construction was completed by one of us [47, 48] following earlier work of Angenent [7] for $$n=1$$ and Koch [40] for $$n>1$$.

The remainder of this manuscript is structured as follows: the Section 2, we rewrite the problem in terms of the relative error, for which most of our analysis is conducted, summarize the spectral theory and introduce some notation. Along with the necessary terminology, Section 3 states our two main dichotomy results. After recalling variants of the Merle–Zaag lemma [42] due to K. Choi with Haslhofer and Hershkovits on the one hand [18] and with Sun on the other [20], our first dichotomy—separating fast and slow convergence—is proved in Section 4, apart from the parabolic regularity estimates which yield a quadratic bound in the relative error for the nonlinearity. These are postponed to Section 5. The remaining dichotomy is established in Section 6.

## Linearized Dynamics and Relative Error

In terms of the relative error $h:=\frac{v-V}{V}$, the dynamics (1.2) take the form

2.1 $$\begin{aligned} \begin{aligned} \partial _t h + L_V h = N (h),\end{aligned}\end{aligned}$$

where $L_V$ is the linear operator relative to *V*,

$$\begin{aligned} \begin{aligned} L_{V} h&= -\frac{1}{ V^{p}} \Delta (hV) - p h\\&= -V^{1-p}\Delta h - 2V^{-p} \nabla V\cdot \nabla h - \left( {p-1}\right) h\\&= -V^{-1-p}\nabla \cdot (V^2 \nabla h) - \left( {p-1}\right) h, \end{aligned} \end{aligned}$$

and *N*(*h*) is the nonlinearity, given by

2.2 $$\begin{aligned} \begin{aligned} N (h)&= (1+h)^p - 1 - p h + \big (1-(1+ h)^{p-1} \big ) \partial _t h .\end{aligned}\end{aligned}$$

Observe that $N(h) = M_V(h)$ for any solution *h* to (2.1), where

2.3 $$\begin{aligned} {M_V} (h) = \frac{1}{(1+h)^{p-1}} \left( (1+h)^p-1-ph\right) + \left( 1- \frac{1}{(1+h)^{p-1}} \right) L_{V}h. \end{aligned}$$

Indeed, solving (2.1) for $\partial _t h$, dividing by the prefactor and shifting the nonlinearities onto the right-hand side yields $N(h) = M_V(h)$. This allows us to exchange temporal for spatial derivatives of *h* in the nonlinearity. Since in most parts of the paper the reference stationary solution is fixed, we will often write $L=L_V$ for notational simplicity.

The relative error and the linear operator are best understood when analyzed in suitable weighted Lebesgue and Sobolev spaces. Given $\sigma >0$ and a positive solution *V* to (1.3), the weighted inner product

$$\begin{aligned} \langle f,g\rangle _{\sigma }= \int _\Omega fg V^\sigma \hbox {d}x \end{aligned}$$

makes $L^2_\sigma =L^2_\sigma (\Omega ):= \{ f:\, \langle f,f\rangle _{\sigma } <\infty \}$ into a Hilbert space. We will occasionally be concerned with more general Lebesgue spaces induced by the norm

$$\begin{aligned} \Vert f\Vert _{L^q_{\sigma }} = \left( \int _{\Omega } |f|^q\, \hbox {d}\mu _{\sigma }\right) ^{\frac{1}{q}}, \end{aligned}$$

where $\hbox {d}\mu _p(x):=V(x)^p \hbox {d}x$. Weighted (homegeneous) Sobolev spaces such as $\dot{H}^1_{\sigma }$ are defined analogously; c.f. (2.4) below.

Multiplication by *V* acts as an isometry between $L^2_{p+1}$ and $L^2_{p-1}$. Under this isometry, the linear operator $L_V + pI$ is unitarily equivalent to an operator $\tilde{L} = V \circ {(L_V + pI)} \circ V^{-1}$ with compact inverse on $L^2_{p-1}$, whose spectral theory, subject to vanishing Dirichlet boundary conditions, was elucidated by Bonforte and Figalli [11]: the corresponding operator $L{=L_V}$ is a self-adjoint semibounded operator on $L^2_{p+1}$;the spectrum of *L* is discrete and the eigenfunctions form a basis of $L^2_{p+1}$. They are critical points for the restriction of the weighted Dirichlet energy

2.4 $$\begin{aligned} E_V(\phi ) = \Vert \phi \Vert _{\dot{H}^1_2}^2 :=\int _\Omega |\nabla \phi |^2 V^2 \hbox {d}x \end{aligned}$$

to that subset of the $L_{p+1}$ unit-sphere for which the boundary trace of $\phi V$ vanishes. Using two nonnegative integers *I* and *K*, let us list the eigenvalues with repetition as

$$\begin{aligned} \begin{aligned}\lambda _{-I} \leqq \ldots \leqq \lambda _{-1}< 0= \lambda _{0}= \ldots = \lambda _{K-1} < \lambda _{K} \leqq \ldots . \end{aligned}\end{aligned}$$

- The integer *I* represents the dimension of the unstable modes of *L* (and coincides with the Morse index of *E*(*v*) from (1.4) at *V*).
- *K* represents the dimension of kernel of *L* and any corresponding eigenfunctions are called Jacobi fields.
- Let us call the eigenfunctions which correspond to $\lambda _{-I}$ to $\lambda _{-1}$ the *unstable modes*, those corresponding to $\lambda _{0}$ to $\lambda _{K-1}$ the *neutral (or central) modes*, and the remaining eigenfunctions (starting with eigenvalue $\lambda _{K}$)the *stable modes.* The corresponding eigenspaces will be denoted by $E_u$, $E_c$ and $E_s$, respectively. They are understood as subspaces of $L^2_{p+1}$, so that $L^2_{p+1} = E_u\oplus E_c \oplus E_s$.
- $\lambda _{{-I}} = 1-p= 1-\frac{1}{m}$ and it is actually simple (a.k.a. multiplicity 1, so $\lambda _{-I}<\lambda _{1-I}$) with corresponding eigenfunction 1 (called the ground state). In the original variables it corresponds to time translation of the solution; the signs $I>0>\lambda _{-I}$ account for the fact that $\tau$-translations of a given solution disappear at different times *T* hence diverge sharply from each other under the rescaling appropriate for one of them.

**Notation.** We finally comment briefly on some notation that we will frequently use throughout this work: We write $a\lesssim b$ if there is a constant *C* such that $a\leqq C b$. The constant *C* may depend on the limiting profile *V*, the domain, and other parameters such as $p=1/m$, but this dependence is continuous under small perturbations of *V* in the relatively-uniform topology generated by (3.6) below, hence *C* may be regarded as being locally independent of *V*. We write $t \gg 1$ to indicate *t* must be sufficiently large.

## Fast Versus Slow Convergence Dichotomies

Recall Bonforte et al.  [12] showed if *V*(*x*) is the limit solution of *v*(*x*, *t*) (see [32]) then the relative error $h(t) = \frac{v(t)}{V} -1$ decays uniformly:

3.1 $$\begin{aligned} \begin{aligned} \Vert h \Vert _{L^\infty (\Omega )} = o(1) \text { as } t\rightarrow \infty . \end{aligned}\end{aligned}$$

In this section we describe two dichotomy theorems which establish that a spectral gap gives the sharp rate of exponential convergence, unless the linearized dynamics has a non-integrable kernel in the refined sense of Definition 3.3. When Definition 3.3 fails to be satisfied, we show the convergence occurs either exponentially at the rate of the spectral gap or no faster that *O*(1/*t*).

### Theorem 3.1

(First dichotomy for asymptotic behavior) For $0<m\in {(m_{1-\frac{n}{2}},1)}=(\frac{n-2}{n+2},1)$, let $\Omega \subset \mathbb {R}^n$ be a smooth bounded domain and $v(x,t) {\geqq 0}$ on $(x,t)\in \Omega \times [0,\infty )$ be a bounded solution to the evolution problem (1.2). Let *V*(*x*) be the classical solution to (1.3) satisfying (1.5). Then there exist positive constants $\varepsilon =\varepsilon (V,p)$ and $C=C(V,p)$ such that whenever $\Vert h(t)\Vert _{L^\infty } \leqq \varepsilon$ for all $t \geqq 0$, exactly one of the following alternatives holds:

1. the relative error $h(t):= \frac{v(t)}{V} -1$ decays algebraically or slower
  3.2 $$\begin{aligned} \begin{aligned} C\left\| h(t) \right\| _{L^\infty } \geqq \left\| h(t) \right\| _{L^2_{p+1}} \geqq (Ct)^{-1} \qquad \forall \ t \gg 1; \end{aligned}\end{aligned}$$
2. the relative error *h* decays exponentially or faster,
  3.3 $$\begin{aligned} \begin{aligned} C^{-1}\left\| h(t) \right\| _{L^2_{p+1}}\leqq \left\| h(t) \right\| _{L^\infty }\leqq C e^{-\lambda t} \Vert h(0)\Vert _{L^2_{p+1}} \quad \forall \ t {\geqq 1, }\end{aligned}\end{aligned}$$
  where $\lambda =\lambda _K>0$ is the first positive eigenvalue of linearized operator *L* in (2.1).
  Moreover, whenever (3.3) holds for some $\lambda \geqq \lambda _K$ and any $C=C(V,p,\lambda )$, if $J \in \textbf{N}_0$ is given such that $\lambda _J< 2\lambda$ and $\lambda _J<\lambda _{J+1} =: \mu$, and $\varphi _0, \varphi _{1}, \dots ,\varphi _J$ are corresponding eigenfunctions chosen in such a way that they can be extended to a complete $L^2_{p+1}$ orthonormal basis, then there are constants $C_i\in \mathbb {R}$ such that $t \geqq 1$ yields
  3.4 $$\begin{aligned} \begin{aligned} \left\| h(t)- \sum _{i=0}^{J} C_{i} e^{-\lambda _i t} \varphi _i\right\| _{L^2_{p+1}} {\leqq \tilde{C}\Vert h(0)\Vert _{L^2_{p+1}} \times } {\left\{ \begin{array}{ll} e^{-2\lambda t} &{} \quad \text{ if } \mu >2\lambda ,\\ te^{-2\lambda t}&{} \quad \text{ if } \mu =2\lambda ,\\ e^{-\mu t} &{} \quad \text{ if } \mu <2\lambda ,\end{array}\right. } \end{aligned}\end{aligned}$$
  for some $\tilde{C}=\tilde{C}(V,p,\lambda ,C)$. Finally, $C_i=0$ for any $\lambda _i \in [0,\lambda )$ so, in particular, for any $i< K$.

### Remark 3.2

1. The result of Bonforte–Figalli in [11] shows (3.3) when $K=0$ (meaning $\ker \, L_V$ is trivial). This assumption prevents the first alternative (3.2).
2. As Bonforte and Figalli argued, we can relax the boundedness assumption on *v* in Theorem 3.1 since initial conditions
  $$\begin{aligned} v_0 \in {\left\{ \begin{array}{ll} \qquad \qquad L^1(\Omega ) &{} \quad \textrm{if}\ m \in {(m_0,1)}=(1-\frac{2}{n},1) \\ \bigcup \limits _{q>\frac{n}{2} (1-m)} L^q(\Omega ) &{} \quad \textrm{if}\ m \in {(m_{1-\frac{n}{2}},m_0]} =(\frac{n-2}{n+2},1-\frac{2}{n}] \end{array}\right. } \end{aligned}$$
  lead to solutions which remain bounded after any short time [28].
3. Due to Bonforte, Grillo and Vazquez result (3.1), the smallness assumption on our initial data is no restriction either.
4. Notice the factor *t* appearing in the bound (3.4) when $\mu =2\lambda$ accounts for the possibility of the kind of eigenvalue resonances described in [7].

As remarked above, triviality of the kernel, $K=0$, implies exponential decay. However, we can also show that in certain situations, exponential decay also occurs when $K>0$. For example, some kernel of *L* can be obtained as a tangent variation among stationary states (as for the non-rotationally symmetric limit states *V* found by Akagi and Kajikiya on thin annuli [4]): if there is a one-parameter family $(V_s)_{s \in (-s_0,s_0)}$ of solutions to (1.3) with $V_0=V$ and $\partial _s V (0)\ne 0$, then $L({\frac{\partial _s V(0)}{V}}) = 0$. If all zero modes are accounted for by such continuous symmetries in the sense of Definition 3.3 below, exponential decay occurs in spite of the fact that $K>0$. To formulate this more precisely, consider the whole family $S \subset H^{1}(\Omega )$ of weak solutions *V* to the Dirichlet problem (1.3), i.e. Sobolev functions for which $\varphi \in C^1(\bar{\Omega })$ implies

3.5 $$\begin{aligned} \int _\Omega \nabla \varphi \cdot \nabla V \hbox {d}x = \int _\Omega \varphi V^p \hbox {d}x. \end{aligned}$$

In Lemma 5.1, we shall eventually infer $S \subset C^{3,\alpha }(\Omega )$ for some $\alpha \in (0,1)$ and that $V/W \in L^\infty (\Omega )$ for all $V,W \in S$. The latter implies that the sets

3.6 $$\begin{aligned} B_r(V) := \{ W \in S :\, \Vert \tfrac{W}{V} -1 \Vert _\infty < r\} \end{aligned}$$

form the base $\{B_r(V)\}_{r>0, V \in S}$ for a topology on *S*, called the *relatively-uniform* topology. We call $V \in S$ an *ordinary limit* if *S* forms a manifold of dimension $K=\dim \ker L_V$ near *V*, which the error relative to *V* embeds differentiably into $L_{p+1}$. More explicitly, call *h* a *stationary relative error* if it is a time-independent solution to (2.1), or equivalently satisfies the nonlinear (singularly) elliptic equation

3.7 $$\begin{aligned} L_V h = M(h),\quad \text{ where } M(h) =(1+h)^p-1-ph; \end{aligned}$$

here $M(h)=N(h) = M_V(h)$ since *h* is stationary.

### Definition 3.3

(*Ordinary limit*) We say that *V* is an *ordinary limit* if there exists a constant $\delta \in (0,1)$, an $L^2_{p+1}$-open neighborhood $\mathcal {U}$ of 0 in $\ker L_V$ and a $C^1$ diffeomorphism

$$\begin{aligned} \Phi _V: (\mathcal {U},\Vert \cdot \Vert _{L^2_{p+1}}) \rightarrow (\{ h\in L^\infty \cap \dot{H}^1_2:\, L_Vh = M(h),\, \Vert h\Vert _{L^{\infty }}\leqq \delta \},\Vert \cdot \Vert _{L^2_{p+1}}), \end{aligned}$$

with the properties that

$$\begin{aligned} \Phi _V(0)=0,\quad (\hbox {d}\Phi _V)_0 = \textrm{id}. \end{aligned}$$

In this definition, the identity map is the one on $\ker L_V$.

The point of this definition is the following theorem:

### Theorem 3.4

(Second dichotomy: convergence to ordinary limits is fast) Under the hypotheses of Theorem 3.1, if a solution to (1.2) converges to an ordinary limit $V \in S$, then convergence takes place exponentially fast: i.e., (3.3) holds.

Here are some preliminary (rather formal) observations.

### Remark 3.5

(Ordinary limits have integrable kernels)

(1) If *h*(*s*) is a smooth curve with $L_Vh(s) = M(h(s))$ and $h(0)=0$, then $\psi = \partial _s h(0) \in \ker L_V$.

(2) If $\psi \in \ker L_V$ consider $h(s) = \Phi _V(s\psi )$ for *s* small. Then

$$\begin{aligned} L_V h(s) = M(h(s)),\quad h(0)=0,\quad \partial _s h(0)=\psi . \end{aligned}$$

This remark shows the kernel of $L_V$ satisfies the next definition if $V \in S$ is an ordinary limit.

### Definition 3.6

(*Integrable kernel*) The kernel of $L_V$ is called *integrable* if for each $\varphi \in \ker L_V$, there is a one-parameter family $\{V_s\}_{s\in (-\varepsilon ,\varepsilon )} \subset S$ of solutions to (1.3), with $V_0=V$ and ${\frac{\partial _s V(0)}{V}} =\varphi$.

In the context of minimal surfaces and geometric evolution equations, analogous concepts of kernel integrability date back at least to Allard–Almgren [3] and Simon [49] respectively. Simon used analyticity to show a converse to Remark 3.5. Inspired by this, it is natural to expect integrability of the kernel of $L_V$ to imply that *V* is an ordinary limit—though we have not verified this in the present context. Note that a limit *V* can fail to be ordinary in one of three ways: either (a) *S* can fail to be a manifold nearby; or (b) *S* can be a manifold locally which the relative error fails to embed differentiably into $L^2(V^{p+1})$; or (c) locally *S* can be a differentiably embedded manifold whose dimension is strictly less than that of $\ker L_V$. It is natural to expect that limits which fail to be ordinary are rare: To the extent that the behaviour of *S* mimics the stratification and singularities of an analytic variety, we imagine that (a) occurs only on a set having local codimension one in *S*. Based on the regularity results we establish below, we are skeptical that (b) ever occurs. And inspired by the genericity results of, e.g., Saut and Teman [50], we conjecture that (c) is non-generic in the sense that there is a dense $G_\delta$ in *S* on which the kernel of the linearized operator has the same dimension as *S*; i.e. the extra zero modes associated with non-ordinary limits are coincidences whose eigenvalues become non-zero upon perturbation. (A weaker conjecture is that a perturbation of $\Omega$ as well as *V* preserving the dimension of *S* is required to restore ordinariness.) It is these expectations that motivate our choice of the term ‘ordinary’.

We finally translate the leading order asymptotics that we found for the relative error back to the original fast diffusion equation (1.1) and its convergence towards the separation-of-variables solution

3.8 $$\begin{aligned} W(x,\tau ) = ((1-m)(T-\tau ))^{\frac{1}{1-m}} V(x)^{\frac{1}{m}}. \end{aligned}$$

### Theorem 3.7

(Quantitative approach to self-similarity in original variables) For $0<m\in ( {m_{1-\frac{n}{2}}},1)$, let $\Omega \subset {\textbf{R}}^n$ be a bounded smooth domain and $w(x,\tau ) {\geqq 0}$ on $(x,\tau )\in \Omega \times [0,\infty )$ be a bounded solution to the evolution (1.1) converging towards the separation-of-variables solution $W(x,\tau )$ given by (3.8). Then there exists $C=C(p,V)$ such that for $\Vert \frac{w^m}{W^m}-1\Vert _{L^\infty (\Omega \times [0,T])}$ and $T-\tau >0$ sufficiently small, after multiplication by $(T-\tau )^{-\frac{1}{1-m}}$ exactly one of the following alternatives holds:

1. the difference $w-W$ decays logarithmically or slower,
  $$\begin{aligned} {(T-\tau )}^{-\frac{1}{1-m}}\Vert w(\tau )-W(\tau ) \Vert _{L^1_1} \geqq \frac{1}{ C \log ^2({1-\frac{\tau }{T}})}; \end{aligned}$$
2. or the difference $w-W$ decays algebraically or faster,
  $$\begin{aligned} {(T-\tau )}^{-\frac{1}{1-m}}\Vert w(\tau )-W(\tau ) \Vert _{L^\infty } {\leqq C {\Vert \frac{w^{m}(0)}{W^{m}(0)}-1 \Vert _{L^2_{p+1} }}} ({1-\frac{\tau }{T}})^{\frac{ m\lambda _{K}}{1-m}}. \end{aligned}$$

### Remark 3.8

Analogous estimates hold true for the relative error $\frac{w(\tau )}{W(\tau )}-1$.

### Proof

(Proof, Case 1) We start with the logarithmic decay estimate for which we assume that the first case in Theorem 3.1 applies. Then

$$\begin{aligned} \frac{1}{t} \lesssim \Vert h(t)\Vert _{L^2_{p+1}} = \Vert v(t) - V\Vert _{L^2_{p-1}} \lesssim \Vert v(t)-V\Vert _{L^1_{p}}^{\frac{1}{2}}, \end{aligned}$$

where we have used in the second inequality that $(v-V)^2 = (v+V)(v-V) = (h+2)V (v-V) \lesssim V |v-V|$ by the uniform boundedness of *h*. Hence, by the definitions of *w* and *W* and the relation between *t* and $\tau$, the latter estimate is equivalent to

$$\begin{aligned} \frac{1}{\log ^2({1-\frac{\tau }{T}})} \lesssim (T-\tau )^{-\frac{m}{1-m}} \Vert w(\tau )^m - W^m(\tau )\Vert _{L^1_{p}}. \end{aligned}$$

We now use the elementary estimate $|a^m-b^m| \leqq m\left( a^{m-1}+b^{m-1}\right) |a-b|$ for $a,b\in {\textbf{R}}_+$ to bound

$$\begin{aligned} |w(\tau )^m-W(\tau )^m|V^{p-1}&\lesssim \left( w(\tau )^{m-1} + W(\tau )^{m-1}\right) V^{p-1} |w(\tau )-W(\tau )|\\&\quad \lesssim (T-\tau )^{-1} \left( \frac{V^{p-1}}{v(t)^{p-1}}+ 1 \right) |w(\tau )-W(\tau )|. \end{aligned}$$

Since $V/v = 1/(h+1)$ is uniformly bounded by hypothesis, the above analysis gives that

$$\begin{aligned} \frac{1}{\log ^2({1-\frac{\tau }{T}})} \lesssim (T-\tau )^{-\frac{1}{1-m}} \Vert w(\tau ) - W(\tau )\Vert _{L^1_{1}}, \end{aligned}$$

as desired.

*Case 2.* We finally convert the exponential decay estimate on the relative error into an estimate for the fast diffusion equation (1.1). From the second case in Theorem 3.1, the definitions of *W* and *w* and the relation between *t* and $\tau$ we infer that

$$\begin{aligned} \Vert h(t)\Vert _{L^{\infty }} \lesssim {\Vert \frac{w^m(0)}{W^m(0)}-1 \Vert _{L^2_{p+1}} } e^{-\lambda _{K} t} \lesssim ({1-\frac{\tau }{T}})^{\frac{ m\lambda _{K}}{1-m}} \end{aligned}$$

We shall now invoke the elementary estimate $|a-1|\lesssim |a^m-1|$ which holds true for *a* close to 1 to conclude a control on the relative error,

$$\begin{aligned} \Vert \frac{w(\tau )}{W(\tau )} -1\Vert _{L^{\infty }} \lesssim {\Vert \frac{w^{m}(0)}{W^{m}(0)}-1 \Vert _{L^2_{p+1}} }( {1-\frac{\tau }{T}})^{\frac{ m\lambda _{K}}{1-m}}, \end{aligned}$$

which yields the desired statement in view of the scaling of *W* and the boundedness of *V*,

$$\begin{aligned} \Vert w(\tau )-W(\tau ) \Vert _{L^{\infty }}&\leqq \Vert W(\tau )\Vert _{L^{\infty }} \Vert \frac{w(\tau )-W(\tau )}{W(\tau )} \Vert _{L^{\infty }}\\&\lesssim (T-\tau )^{\frac{1}{1-m}} \Vert \frac{w(\tau )}{W(\tau )}-1 \Vert _{L^{\infty }}. \end{aligned}$$

This concludes the proof of the theorem.

## Proof of First Dichotomy (Apart from Smoothing Estimates)

We start with an $L^2$ semigroup estimate which is at the heart of our analysis.

### Lemma 4.1

(Energy growth control under nonlinear evolution) Let *h* be a solution to the nonlinear equation (2.1)–(2.2) with initial datum $h_{0}\in L^2_{p+1}$, and assume that $\Vert h\Vert _{L^{\infty }} \leqq \varepsilon$ for some $\varepsilon >0$. Then there exists $C>0$ such that for $\varepsilon >0$ small enough and all $t>0$, the following holds:

$$\begin{aligned} \Vert h(t)\Vert _{L^2_{p+1}}^2 +\int _0^t \Vert \nabla h\Vert _{L^2_2}^2\, \textrm{d}t\le e^{Ct} \Vert h_0\Vert _{L^2_{p+1}}^2. \end{aligned}$$

### Proof

It will be convenient to consider the purely spatial form (2.3) of the nonlinearity $N(u) =M_V(u)$ when working with (2.1)–(2.2). Then setting $\hbox {d}\mu _p(x):=V(x)^p \hbox {d}x$, testing (2.1) with $h V^{p+1}$ and integrating by parts, we arrive at the identity

$$\begin{aligned}&\frac{1}{2}\frac{\hbox {d}}{\hbox {d}t} \int h^2 \, \hbox {d}\mu _{p+1} + \int |\nabla h|^2\, \hbox {d}\mu _2 \\&\quad = (p-1) \int h^2\, \hbox {d}\mu _{p+1} + \int \frac{h}{(1+h)^{p-1}}\left( (1+h)^p-1-ph\right) \, \hbox {d}\mu _{p+1} \\&\qquad +\int \left( \left( \frac{1}{1+h}\right) ^{p-1}-1 \right) |\nabla h|^2\, \hbox {d}\mu _2 + (p-1) \int h \left( \frac{1}{1+h}\right) ^{p} |\nabla h|^2\,\hbox {d}\mu _2\\&\qquad +(p-1) \int \left( \left( \frac{1}{1+h}\right) ^{p-1}-1\right) h^2\, \hbox {d}\mu _{p+1}. \end{aligned}$$

Because $|h|\leqq \varepsilon <1$, the right-hand side can be controlled by quadratic expressions, more precisely,

$$\begin{aligned} \frac{\hbox {d}}{\hbox {d}t} \int h^2 \, \hbox {d}\mu _{p+1} + \int |\nabla h|^2 \, \hbox {d}\mu _2 \lesssim \int h^2\, \hbox {d}\mu _{p+1}+\varepsilon \int |\nabla h|^2 \, \hbox {d}\mu _2. \end{aligned}$$

If $\varepsilon$ is sufficiently small, the gradient terms can be absorved into the right hand side, and the resulting estimate can be solved with the help of a standard Gronwall argument. This proves the lemma.

The proof of our first dichotomy, Theorem 3.1, is based on two main ingredients. On the one hand, our argument will rely on a fundamental dynamical systems result that is due to Merle and Zaag [42], and which in turn improves on an earlier related result by Filippas and Kohn [31]. On the other hand, we have to exploit some smoothing properties of the parabolic equation.

The Merle–Zaag lemma is concerned with a system of weakly coupled first order ordinary differential equations featuring stable, neutral and unstable solutions. It states that under the assumption that the unstable modes fail to grow, the long time asymptotics are dominated by precisely one of the other two modes. This lemma provides an effective way to extract the quantized behaviour of the solution prescribed by the discrete spectrum of its limit. It plays a pivotal role in recent progress on classifications of ancient solutions (solutions defined for $(-\infty ,T]$) to parabolic equations [1, 2, 9, 19] and entire solutions to elliptic equations [17] arising from geometry. In classifications of ancient flows, the lemma is applied backward in time (i.e. $t=-s$). One advantage in backward problems is that there are only finitely many stable eigenfunctions (imagine, for instance, the laplacian $\Delta$ has only finitely many positive eigenfunctions); this makes the classifications possible. Meanwhile in the forward problem, there are infinitely many stable eigenfunctions. The lemma is used to investigate the asymptotic behavior of solutions. To obtain the stated dependencies of the constants in our first dichotomy requires a refinement of the Merle–Zaag Lemma due to K. Choi et al.

### Lemma 4.2

(Choi–Haslhofer–Hershkovits refinement [18, Lemma 4.6]) Let *X*(*s*), *Y*(*s*), and *Z*(*s*) be non-negative absolutely continuous functions on $[0,\infty )$ satisfying $X+Y+Z >0$,

$$\begin{aligned} \begin{aligned} \frac{\textrm{d}x}{\textrm{d}s} -X&\geqq -\varepsilon (Y + Z), \\ |\frac{\textrm{d}Y}{\textrm{d}s}|&\leqq \varepsilon (X+Y+Z ), \text{ and }\\ \frac{\textrm{d}Z}{\textrm{d}s} + Z&\leqq \varepsilon (X+Y), \end{aligned} \end{aligned}$$

for each $\varepsilon \in (0, \frac{1}{100})$ and a.e. $s\in [s_0(\varepsilon ),\infty )$. If

$$\begin{aligned} \lim _{s\rightarrow \infty } (X + Y + Z)(s) = 0, \end{aligned}$$

then $X \leqq 2\varepsilon (Y+Z)$ for $s\geqq s_0(\varepsilon )$ and either

$$\begin{aligned} X(s)+Z(s)= o(Y(s)) \text { as } s\rightarrow \infty \end{aligned}$$

or

$$\begin{aligned} X(s)+Y(s) \leqq 100 \varepsilon Z(s) \text { for } s\geqq s_0(\varepsilon ). \end{aligned}$$

### Proof

A proof is given in Appendix B of [19].

For later use we also recall a quantitative adaptation of the Merle Zaag lemma to a compact time interval proved by Choi and Sun in [20]. In Sect. 6 their result will be motivated and used to prove our second dichotomy.

### Lemma 4.3

(Choi–Sun refinement [20, Lemma B.2]) Suppose *X*(*s*), *Y*(*s*), and *Z*(*s*) are non-negative absolutely continuous functions on some interval $[-L,L]$ such that $0<X+Y+Z< \eta$ for some $\eta >0$. Suppose that there exist two constants $\sigma >0$ and $\Lambda >0$ such that

$$\begin{aligned} \begin{aligned} \frac{\textrm{d}X}{\textrm{d}s} - \Lambda X&\geqq - \sigma (Y+Z), \\ |\frac{\textrm{d}Y}{\textrm{d}s}|&\leqq \sigma (X+Y+Z),\\ \frac{\textrm{d}Z}{\textrm{d}s} + \Lambda Z&\leqq \sigma (X+Y) , \end{aligned}\end{aligned}$$

for any $s\in [-L,L]$. Then there exists $\sigma _0=\sigma _0(\Lambda )$ such that if $0<\sigma <\sigma _0$ it holds that

$$\begin{aligned} \begin{aligned} X+Z \leqq \frac{8\sigma }{\Lambda } Y + 4\eta e^{-\frac{\Lambda L}{4}} \text{ for } \text{ any } s\in [-L/2,L/2].\end{aligned}\end{aligned}$$

The next proposition provides the crucial control that we need to estimate the nonlinear terms in the relative error dynamics (2.1) quadratically.

### Proposition 4.4

(Spatially uniform control of time derivatives) Let $k\in \textbf{N}_0$ and $t>0$ fixed. Then if $\Vert h\Vert _{L^{\infty }}\leqq \varepsilon$ with $\varepsilon$ sufficiently small, there exists a constant $C=C(t,k{,m,V})$ such that

$$\begin{aligned} \Vert \partial _t^{k} h(t) \Vert _{L^{\infty }} \leqq C \Vert h_0\Vert _{L^2_{p+1}}. \end{aligned}$$

We postpone the proof and a discussion of this proposition to the next subsection and show first how to deduce our first dichotomy from Lemmas 4.1–4.2 and Proposition 4.4.

### Proof of Theorem 3.1

In this proof, for the sake of convention, we omit the subscript $L^2_{p+1}=L^2(V^{p+1})$ and use $\Vert f \Vert : = \Vert f \Vert _{L^2_{p+1}}$. Positive constants $\varepsilon =\varepsilon (V,p)$ and $C=C(V,p)$ are not fixed yet, $\varepsilon$ may become smaller, and *C* may become larger until they are fixed.

Let us denote by $P_s$, $P_c$ and $P_u$ the orthogonal projections of $L^2_{p+1}$ onto the stable, center, and unstable eigenspaces $E_s$, $E_c$ and $E_u$, respectively. Moreover, we write $h_s=P_sh$, $h_c=P_ch$, and $h_u=P_uh$ for the projected solutions. A straightforward computation reveals that

4.1 $$\begin{aligned} \begin{aligned} \frac{\hbox {d}}{\hbox {d}t} \Vert h_u\Vert _{ }&\geqq -\lambda _{{-1}} \Vert h_u \Vert _{ }- \Vert {N}(h) \Vert _{ },\\ \left| \frac{\hbox {d}}{\hbox {d}t} \Vert h_c \Vert _{ } \right|&\leqq \Vert {N}(h) \Vert _{ }, \\ \frac{\hbox {d}}{\hbox {d}t}\Vert h_s \Vert _{ }&\leqq -\lambda _{{K}} \Vert h_s \Vert _{ } + \Vert {N}(h) \Vert _{ } , \end{aligned}\end{aligned}$$

where we recall $\lambda _{{-1}}$ and $\lambda _{{K}}$ are the negative and positive eigenvalues of $L {=L_V}$ closest to zero. As an example, for the case of the evolution on the stable subspace, we observe that

4.2 $$\begin{aligned} \partial _t h_s + L h_s = P_s{N}(h), \end{aligned}$$

and testing with $h_s V^{p+1}$ gives

$$\begin{aligned} \frac{1}{2}\frac{\hbox {d}}{\hbox {d}t} \Vert h_s\Vert ^2 + \langle h_s, Lh_s\rangle = \langle h_s,N(h)\rangle . \end{aligned}$$

The third estimate in (4.1) now results from the lower bound on the stable eigenvalues and the Cauchy–Schwarz inequality. The first and the second estimates are derived analogously.

In order to estimate the nonlinearities in (4.1), we note that for $|h| \leqq \varepsilon$, (provided that $\varepsilon$ is sufficiently small) there is $C_1=C_1(p)$ such that

4.3 $$\begin{aligned} \begin{aligned} |{N}(h)|&\leqq C_1 |h| \left( \left| h\right| + \left| \partial _t h\right| \right) \\ {}&{\leqq C_1 |h| \left( \left| h\right| + C\left\| h(t-1) \right\| \right) }, \end{aligned}\end{aligned}$$

by Taylor expansion followed by the smoothing estimates of Proposition 4.4 for $t \geqq 1$. By virtue of Bonforte et al. [12] uniform bound on the relative error (3.1), for *any* small ${\hat{\varepsilon }}>0$ there exists a time $t_0(\hat{\varepsilon })$ such that

4.4 $$\begin{aligned} \begin{aligned} \Vert {N}(h(t))\Vert _{ } \leqq {\hat{\varepsilon }} \Vert h(t)\Vert _{ } \text { for any }t\geqq t_0(\hat{\varepsilon }).\end{aligned}\end{aligned}$$

Plugging this estimate into (4.1), we observe that if $h\ne 0$, then $h_s$, $h_c$ and $h_u$ satisfy the hypothesis Lemma 4.2 with $s=\lambda t$ where $\lambda := \frac{1}{2} \min (|\lambda _{-1}|, |\lambda _K|)$. Therefore, unless *h* is stationary (and thus trivial), either

4.5 $$\begin{aligned} \begin{aligned} \Vert h_u\Vert _{ }+\Vert h_s\Vert _{ } = o(\Vert h_c\Vert _{ }) \end{aligned}\end{aligned}$$

or

4.6 $$\begin{aligned} \begin{aligned} \Vert h_u\Vert _{ }+\Vert h_c\Vert _{ } \leqq \frac{100 \hat{\varepsilon }}{\lambda } \Vert h_s \Vert \text { for } t\geqq t_0(\hat{\varepsilon }) .\end{aligned}\end{aligned}$$

Note in particular, the second alternative (4.6) implies $\Vert h_u\Vert + \Vert h_c \Vert = o (\Vert h_s\Vert )$. We discuss the implications of each case individually, starting from the latter case (4.6).

**Case 2.** First, by choosing $\varepsilon =\varepsilon (V,p)$ sufficiently small, we may assume $\Vert h_u\Vert + \Vert h_c \Vert \leqq \frac{1}{2} \Vert h_s \Vert$ for all $t \geqq 1$. Here, note that $t\geqq 1$ is needed as Proposition 4.4 is applied to estimate *N*(*h*). Moreover, by the same argument we used to derive (4.4), there is $C_2=C_2(V,p)$ such that

$$\begin{aligned} \Vert N(h(t))\Vert \leqq C_2 \varepsilon \Vert h(t)\Vert \text { for } t\geqq 1. \end{aligned}$$

Combining the above, the estimate of the nonlinearity (4.4) becomes

$$\begin{aligned} \Vert N(h(t))\Vert \leqq C_2 {\varepsilon } \Vert h(t)\Vert \leqq 2C_2 \varepsilon \Vert h_s(t)\Vert , \end{aligned}$$

for $t\geqq 1$. The third estimate in (4.1) thus turns into the differential inequality

$$\begin{aligned} \frac{\hbox {d}}{\hbox {d}t} \Vert h_s\Vert \leqq - (\lambda _{K}-2C_2\varepsilon ) \Vert h_s\Vert , \end{aligned}$$

for $t\geqq 1$, which yields via a Gronwall argument

$$\begin{aligned} \Vert h_s(t)\Vert \leqq e^{-(\lambda _{K}-2C_2\varepsilon ) (t-1) } \Vert h_s(1)\Vert . \end{aligned}$$

By another application of (4.6) and the semigroup estimate from Lemma 4.1, this estimate can be translated into the full solution

4.7 $$\begin{aligned} \Vert h(t)\Vert \lesssim e^{-(\lambda _{K}-2C_2 \varepsilon ) t} \Vert h_0\Vert . \end{aligned}$$

To improve this inequality by removing the $\varepsilon$, fix $\varepsilon$ small so that $2C_ 2 \varepsilon <{\frac{1}{2}}\lambda _{K}$, and refine the estimate of the nonlinearity with the help of the quadratic bound (4.3), the smoothing estimates from Proposition 4.4 and the previous estimate (4.7),

$$\begin{aligned} \Vert N(h(t))\Vert&\lesssim \left( \Vert h(t)\Vert _{L^{\infty }} + \Vert \partial _t h(t)\Vert _{L^{\infty }}\right) \Vert h(t)\Vert \\&\lesssim \Vert h(t-1)\Vert \Vert h(t)\Vert \\&\lesssim e^{-2(\lambda _{K}-2C_2 \varepsilon ) t} \Vert h_0\Vert ^2, \end{aligned}$$

for $t\geqq 1$. Substitution into the third estimate of (4.1) finally establishes a differential inequality, which yields

$$\begin{aligned} \Vert h(t)\Vert \lesssim e^{-\lambda _{K} t} \Vert h_0\Vert , \end{aligned}$$

via (4.6), for all $t \geqq 0$ (recalling $\Vert h_1\Vert \lesssim \Vert h_0\Vert$ from Lemma 4.1). By another application of Proposition 4.4, this estimate can be upgraded towards a uniform convergence result,

$$\begin{aligned} \begin{aligned} \Vert h(t)\Vert _{L^{\infty }} \lesssim e^{-\lambda _{K} t} \Vert h_0\Vert , \end{aligned}\end{aligned}$$

for $t\geqq 1$. Having established (3.3), it remains to identify the leading order expansion (3.4). Let us begin by noting that whenever (3.3) holds for some $\lambda \geqq \lambda _K$ and any *C*, repeating the previous estimate on the nonlinearity yields the improvement

4.8 $$\begin{aligned} { \Vert N(h(t))\Vert {\leqq \tilde{C}} e^{-2\lambda t} \Vert h_0\Vert ^2} \end{aligned}$$

for $t \geqq 1$, where $\tilde{C}= \tilde{C}(V,p,\lambda , C)$. Since the nonlinearity is quadratic, modes corresponding to eigenvalues in the interval $[0,2\lambda )$ are accessible via a simple ODE argument. For any *i*, the projection $y_i = \langle h,\varphi _i\rangle$ satisfies the differential inequality

$$\begin{aligned} \left| \frac{\hbox {d}}{\hbox {d}t} y_i+ \lambda _i y_i \right| \leqq \Vert {N}(h) \Vert _{ } , \end{aligned}$$

which can be rewritten as

$$\begin{aligned} \left| \frac{\hbox {d}}{\hbox {d}t}(e^{\lambda _it}y_i)\right| \lesssim e^{-(2\lambda -\lambda _i)t}, \end{aligned}$$

as a consequence of (4.8). Integration in time thus yields for any $T\geqq t {\geqq 1}$ that

$$\begin{aligned} \left| e^{\lambda _i t}y_i(t) - e^{\lambda _i T}y_i(T)\right| \lesssim e^{-(2\lambda - \lambda _i)t}, \end{aligned}$$

where we have used the fact that $\lambda _i<2\lambda$. This bounds implies that $T \mapsto e^{\lambda _i T }y_i(T)$ is a Cauchy sequence, so that, sending $T\rightarrow \infty$, we find

4.9 $$\begin{aligned} \begin{aligned} \langle h(t),\varphi _i\rangle = y_i(t) = C_i e^{-\lambda _i t} + O(e^{-2\lambda t} ) \end{aligned}\end{aligned}$$

for some $$C_i\in {\textbf{R}}$$.

We now estimate with the help of the decomposition $h = h_s+h_c+h_u$, the triangle inequality and the fact that the eigenfunctions $\varphi _{0},\dots ,\varphi _J$ are orthonormal that

4.10 $$\begin{aligned} \Vert h - \sum _{i=0}^J C_i e^{-\lambda _i t} \varphi _i\Vert \leqq \sum _{i= 0}^J |y_i - C_i e^{-t\lambda _i}| + \Vert h_s - \sum _{i=0}^J y_i \varphi _i\Vert + \Vert h_c\Vert + \Vert h_u\Vert . \end{aligned}$$

We have just seen that the first term on the right-hand side is $\lesssim e^{-2\lambda t}$. Recalling the spectral decomposition of *L* and (4.2), the next contribution, $z=\Vert h_s - \sum _{i=1}^J y_i \varphi _i\Vert$ satisfies the differential inequality

4.11 $$\begin{aligned} \frac{\hbox {d}}{\hbox {d}t} z + \mu z \leqq \Vert {N}(h) \Vert _{ }. \end{aligned}$$

Similarly to the argumentation above, we rewrite (4.11) as

$$\begin{aligned} \frac{\hbox {d}}{\hbox {d}t}(e^{\mu t}z) \lesssim e^{-(2\lambda -\mu )t}, \end{aligned}$$

and deduce that

4.12 $$\begin{aligned} z(t) \lesssim {\left\{ \begin{array}{ll} e^{-2\lambda t} &{} \quad \text{ if } \mu >2\lambda ,\\ te^{-2\lambda t}&{} \quad \text{ if } \mu =2\lambda ,\\ e^{-\mu t} &{}\text{ if } \mu <2\lambda ,\end{array}\right. } \end{aligned}$$

via integration.

Finally, regarding the remaining terms in (4.10), the first two estimates in (4.1) imply that

$$\begin{aligned} \frac{\hbox {d}}{\hbox {d}t} \Vert h_c\Vert +\frac{\hbox {d}}{\hbox {d}t} \Vert h_u\Vert > rsim - e^{-2\lambda t}, \end{aligned}$$

and thus, an integration from *t* to $\infty$ gives, thanks to the fact that $\Vert h_c\Vert _{ } +\Vert h_u\Vert _{ } \rightarrow 0$ as $t\rightarrow \infty$,

4.13 $$\begin{aligned} \begin{aligned} \Vert h_c(t)\Vert _{ } +\Vert h_u(t)\Vert _{ } \lesssim e^{-2\lambda t} .\end{aligned}\end{aligned}$$

Therefore, substituting (4.9), (4.12), and (4.13) into (4.10), we deduce that

$$\begin{aligned} \begin{aligned} \left\| h(t)- \sum _{i=0}^{J} C_{i} \varphi _i e^{-\lambda _i t} \right\| _{ } \lesssim {\left\{ \begin{array}{ll} e^{-2\lambda t} &{} \quad \text{ if } \mu >2\lambda ,\\ te^{-2\lambda t}&{} \quad \text{ if } \mu =2\lambda ,\\ e^{-\mu t} &{} \quad \text{ if } \mu <2\lambda \end{array}\right. } \end{aligned}\end{aligned}$$

as desired. If $C_i\ne 0$ for some $\lambda _i \in [0,\lambda )$, then (3.4) contradicts (3.3); thus $\lambda _i \geqq \lambda$ as asserted.

**Case 1.** In case the neutral modes are dominating (4.5), an estimate analogously to the one in Case 2 above gives rise to the bound

$$\begin{aligned} \begin{aligned} \Vert {N}(h(t))\Vert _{ } \leqq 2C_2\varepsilon \Vert h_c(t)\Vert , \end{aligned}\end{aligned}$$

provided that $t\geqq t_c$ for some $t_c$ large enough. By plugging this into the middle equation of (4.1), we find that

$$\begin{aligned} \frac{\hbox {d}}{\hbox {d}t} \Vert h_c\Vert \geqq -2C_2\varepsilon \Vert h_c\Vert , \end{aligned}$$

and thus, via (4.5),

$$\begin{aligned} \begin{aligned} \Vert h(t+1)\Vert \geqq \Vert h_c(t+1)\Vert _{ } > rsim \Vert h_c(t)\Vert _{ } > rsim \Vert h(t)\Vert \end{aligned}\end{aligned}$$

for any $t\geqq t_c$. With this information at hand, we may reconsider our previous bound on the nonlinearity. This time, making use of the pointwise estimate in (4.3) and the smoothing properties from Proposition 4.4, we have that

$$\begin{aligned} \Vert N(h(t))\Vert \lesssim \Vert h(t-1)\Vert \Vert h(t)\Vert \lesssim \Vert h(t)\Vert ^2 \lesssim \Vert h_c(t)\Vert ^2, \end{aligned}$$

for any $t\geqq t_c$, for some (possibly larger) $t_c$. The second estimate in (4.1) now turns into the growth condition

$$\begin{aligned} \frac{\hbox {d}}{\hbox {d}t} \Vert h_c(t)\Vert > rsim - \Vert h_c(t)\Vert ^2, \end{aligned}$$

which yields the lower bound

$$\begin{aligned} \Vert h(t)\Vert \geqq \Vert h_c(t)\Vert > rsim \frac{1}{t}, \end{aligned}$$

if $t\geqq t_c$ for some $t_c$.

This concludes the proof of Theorem 3.1.

## Smoothing Estimates

In this section, we use parabolic regularity techniques to prove Proposition 4.4. We remark that optimal (boundary) regularity estimates were derived recently by Jin and Xiong for the rescaled solution *v* of (1.2) rather than the relative error *h* [35]. However, from Theorem 5.1 in their paper we easily infer that

5.1 $$\begin{aligned} \partial _t^k h\in C^{0}((\tau ,\infty )\times \Omega ) \end{aligned}$$

for any $k\in \textbf{N}$ and $\tau >0$. This insight will simplify the derivation of our regularity estimates substantially.

Smoothing features are typical for parabolic equations and they remain true in the time and tangential directios for the singular parabolic equation under consideration; see (5.1) and Corollary 5.12. In transversal direction, regularity is limited (if *p* is not an integer) [35]. Indeed, simple scaling arguments for the elliptic problem suggest that $V(x)\sim a{{\,\textrm{dist}\,}}(x,\partial \Omega ) + b {{\,\textrm{dist}\,}}(x,\partial \Omega )^{p+2}$ close to the boundary, and the same behavior can be expected for the parabolic problem (1.2).

For deriving the smoothing estimates in Proposition 4.4, we notice that the leading order contribution in the nonlinearity (2.2) is of the order $|h||\partial _t h| \lesssim \varepsilon |\partial _t h|$, and plays thus the role of a perturbation term in regularity estimates. Moreover, the equation is invariant under differentiation in time and in tangential coordinates near the domain boundary (at least with regard to the leading order contributions), and thus, regularity in these variables is propagated and even further increased by parabolicity. We deal with higher-order derivatives of the nonlinearity by applying suitable interpolations, so that eventually, derivatives of the nonlinearity will play the role of perturbations similarly to the nonlinearity itself as discussed above. Of course, smoothing proceeds instantaneously but not uniformly in time. For this reason, the estimates in or behind Proposition 4.4, Equation (5.1) or Corollary 5.12 deteriorate as $t\rightarrow 0$.

Before addressing the dynamical problem, we need to estimate the first three derivatives for weak solutions of the nonlinear elliptic problem (1.3), starting from the known result (5.2). Later we’ll see that higher-order tangential derivatives of this solution can also be estimated near the domain boundary.

### Lemma 5.1

(Regularity of asymptotic profile) For any $\alpha \in (0,1)$ with $\alpha \leqq p-1$, if $V \in H^1(\Omega )$ satisfies (3.5) for all $\varphi \in C^1(\bar{\Omega })$, then $V\in C^{3,\alpha }(\Omega )$ and, for any $x\in \Omega$,

5.2 $$\begin{aligned} {{\,\textrm{dist}\,}}(x,\partial \Omega ) \lesssim V(x) \lesssim {{\,\textrm{dist}\,}}(x,\partial \Omega ), \end{aligned}$$

5.3 $$\begin{aligned} \text{ and }\ |\nabla V(x)|, |\nabla ^2 V(x)|, |\nabla ^3 V(x)|\lesssim 1 \end{aligned}$$

hold. Furthermore, there exists an $r > rsim 1$ such that

5.4 $$\begin{aligned} |\nabla V(x)| > rsim 1 \end{aligned}$$

for any $x\in \Omega$ with ${{\,\textrm{dist}\,}}(x,\partial \Omega )\leqq r$.

### Proof

For a fixed $V\in S$, the first estimate is established, for instance, in Theorem 1.1 in [28] (on the level of the evolutionary problem) or Theorem 5.9 in [13]; the form of (5.2) makes it clear that the constants depend continuously on *V* in the relatively-uniform topology on *S*. The second and the third estimate follow from maximal regularity estimates and Sobolev embeddings. Indeed, since $V\in L^{\infty }(\Omega )$ thanks to (5.2), we must have that $V\in W^{2,q}(\Omega )$ for any $q\in (1,\infty )$ based on Calderón–Zygmund estimates for the elliptic problem (1.3), see, e.g., Chapter 11 in [41], and thus $V\in C^{1,\alpha }(\bar{\Omega })$ for any $\alpha \in (0,1)$ by Sobolev embeddings. It follows that $\partial _i V^p = pV^{p-1}\partial _i V\in C^{0,{\alpha }}(\bar{\Omega })$ provided that $\alpha \leqq p-1$, and thus one spatial derivative of the equation shows $V\in C^{3,{\alpha }}(\bar{\Omega })$ by Schauder estimates, see, e.g., Chapter 6 in [34].

The statement in (5.4) is a consequence of the above. Indeed, according to (5.2), at the boundary *V* grows linearly in the direction of the inner normal. By the estimates (5.3), we must thus have (5.4) in a neighborhood of the boundary.

Our first step in the derivation of the smoothing estimates is a maximal regularity estimate for the linearization of (2.1). More precisely, we consider the inhomogeneous linear equation

5.5 $$\begin{aligned} \partial _t h - V^{-1-p}\nabla \cdot (V^2 \nabla h) = (p-1)h + f, \end{aligned}$$

with zero initial data. For general initial data $h_0\in L^2_{p+1}$ and inhomogeneities $f\in L^1((0,T);L^2_{p+1})$, a solution is always understood in the weak sense. A *weak solution* of (5.5) refers to a function $h\in L^{\infty }((0,T);L^2_{p+1}) \cap L^2((0,T);\dot{H}^1_2)$ in the spaces provided by Lemma 4.1 such that

5.6 $$\begin{aligned} \begin{aligned}&-\iint _{(0,T)\times \Omega } {h} \partial _t \varphi \, \hbox {d}\mu _{p+1}\hbox {d}t + \iint _{(0,T)\times \Omega } \nabla \varphi \cdot \nabla h\, \hbox {d}\mu _2\hbox {d}t \\&= \iint _{(0,T)\times \Omega } ((p-1)h+f) \varphi \hbox {d}\mu _{p+1}\hbox {d}t + \int _{\Omega } \left. \varphi \right| _{t=0}h_0\, \hbox {d}\mu _{p+1} \end{aligned}\end{aligned}$$

for any $\varphi \in C_{c}^1([0,T)\times \bar{\Omega })$ of compact support. It should be stressed that we do not impose spatial boundary conditions on *h* in the parabolic problem (5.5), which turns out to be well-posed (only) in this case. This is a consequence of the observation that by (formally) integrating by parts in the gradient term in the weak formulation (5.6), the boundary term vanishes thanks to the Dirichlet boundary conditions satisfied by *V*, see (1.3).

Existence of weak solutions can be derived via standard methods, for instance, via Galerkin approximations based on an $L^2_{p+1}$ orthonormal basis consisting of eigenfunctions of the linear operator $L{=L_V}$. Moreover, from standard energy estimates (derived similarly to those in Lemma 4.1), we infer the uniqueness of weak solutions.

What is a crucial tool in our theory is a maximal regularity estimate for the linear equation (5.5), that we consider, for convenience, with $L^2_{2p}$ inhomogenity and zero initial data, see Proposition 5.5 below.

In the interior of $\Omega$, maximal regularity for the parabolic problem (5.5) follows by standard theory, see, e.g., Chapter 7.1 in [30], because the diffusivity coefficients are strictly positive in the interior as a consequence of (5.2). We shall thus focus on the boundary from here on and we fix $x_0\in \partial \Omega$. Let $\eta$ denote a cut-off function on ${\textbf{R}}^n$ interpolating smoothly between $\eta =1$ in $B_r(x_0)$ and $\eta = 0$ outside of $B_{2r}(x_0)$.

A short computation reveals that the localized solution $H = \eta h$ satisfies the problem

5.7 $$\begin{aligned} \partial _t H - V^{-1-p}\nabla \cdot (V^2\nabla H) = F+G, \end{aligned}$$

where *F* and *G* are given by

$$\begin{aligned} F = \eta f,\quad G = -2V^{1-p} \nabla \eta \cdot \nabla h - V^{1-p} h \Delta \eta - 2 V^{-p} h \nabla V \cdot \nabla \eta . \end{aligned}$$

The lemma guarantees that *G* belongs to $L^2(L^2_{2p})$ provided that $h\in L^2(L^2_{p+1})\cap L^2( \dot{H}^1_2)$, which is assumed for our weak solutions.

### Lemma 5.2

(Weighted Poincaré/Hardy type inequality) All $h\in L^2_{p+1}\cap \dot{H}^1_2$ satisfy

$$\begin{aligned} \Vert h\Vert _{L^2} \lesssim \Vert h\Vert _{L^2_{p+1}} + \Vert \nabla h\Vert _{L^2_2}. \end{aligned}$$

In particular, it holds that

$$\begin{aligned} \Vert h\Vert _{L^2_{p+1}} \lesssim \Vert h\Vert _{L^2_{2p}} + \Vert \nabla h\Vert _{L^2_2}. \end{aligned}$$

The proof of the first statement is based on an interpolation argument and the properties of the limit *V*. The latter then follows via Hölder’s and Young’s estimate.

### Proof

We start considering the first estimate. Because $C^{\infty }(\bar{\Omega })$ is dense in $L^2_{p+1}\cap \dot{H}^1_2$, cf. Lemma 2 in [47], it is enough to establish the estimate for smooth functions. We first notice that an integration by parts and the defining properties of *V* in (1.3) yield

$$\begin{aligned} \int h^2 |\nabla V|^2 \, \hbox {d}x&= - \int h^2 V \Delta V\, \hbox {d}x - 2 \int h\nabla h\cdot V\nabla V\, \hbox {d}x\\&= \int h^2 V^{p+1}\, \hbox {d}x - 2 \int h\nabla h\cdot V\nabla V\, \hbox {d}x. \end{aligned}$$

Making use of the elementary inequality $ab \leqq a^2 + b^2/4$ thus gives that

$$\begin{aligned} \Vert h |\nabla V| \Vert _{L^2} \lesssim \Vert h\Vert _{L^2_{p+1}} + \Vert \nabla h\Vert _{L^2_2}. \end{aligned}$$

The first statement of the lemma is now a consequence of the fact that $1\lesssim V^{p+1} + |\nabla V|$, which holds true thanks to Lemma 5.1.

For the second statement, we apply Hölder’s inequality, and the previous bound to estimate

$$\begin{aligned} \Vert h\Vert _{L^2_{p+1}} \leqq \Vert h\Vert _{L^2_{2p}}^{\frac{p+1}{2p}} \Vert h\Vert _{L^2}^{\frac{p-1}{2p}} \lesssim \Vert h\Vert _{L^2_{2p}}^{\frac{p+1}{2p}} \Vert h\Vert _{L^2_{p+1}}^{\frac{p-1}{2p}} + \Vert h\Vert _{L^2_{2p}}^{\frac{p+1}{2p}} \Vert \nabla h\Vert _{L^2_{2}}^{\frac{p-1}{2p}}. \end{aligned}$$

The desired result is then a consequence of Young’s inequality $ab \lesssim a^q + b^{q'}$ for any Hölder conjugates *q* and $q'$.

We shall now flatten the boundary. Upon a rotation of the coordinate system, we may assume that the boundary inside $B_r(x_0)$ can be written as a graph of a function $\gamma$, for instance,

$$\begin{aligned} \Omega \cap B_{2r}(x_0) = \left\{ x=(x',x_n)\in B_{2r}(x_0):\, x_n>\gamma (x')\right\} . \end{aligned}$$

We set $\hat{x} = \phi (x) =(x',x_n-\gamma (x'))$, which defines a diffeomorphism in the support of $\eta$, and maps the boundary $\partial \Omega$ into the hypersurface ${\textbf{R}}^{n-1}\times \{0\}$. In terms of $\hat{H}(t,\hat{x}) = H(t,x)$, $\hat{V}(\hat{x}) = V(x)$, $\hat{F}(t,\hat{x}) = F(t,x)$, and $\hat{G} (t,\hat{x}) = G(t,x)$ the localized equation (5.7) becomes

5.8 $$\begin{aligned} \partial _t \hat{H}- \hat{V}^{-1-p}\hat{\nabla }\cdot (\hat{V}^2 A\hat{\nabla }\hat{H}) = \hat{F} + \hat{G}, \end{aligned}$$

where

$$\begin{aligned} A = \left( \begin{array}{c|c} I &{} -\nabla '\gamma \\ \hline -(\nabla '\gamma )^T &{} 1+|\nabla '\gamma |^2\end{array}\right) . \end{aligned}$$

Notice that the transformed equation (5.8) has to be considered on the halfspace ${\textbf{R}}^n_+$.

The advantage of (5.8) over the (5.5) is that in the new variables, the weight and its tangential derivatives can be estimated by the distance to the flattened boundary.

### Lemma 5.3

(Derivatives of asymptotic profile parallel to flattened boundary) For any $\hat{x} \in \phi (\Omega \cap B_{{2}r}(x_0))$ and $k \in \textbf{N}$, both

$$\begin{aligned} \hat{x}_n \lesssim \hat{V}(\hat{x}) \lesssim \hat{x}_n \end{aligned}$$

and

5.9 $$\begin{aligned} |D^k_{\hat{x}'} \hat{V}(\hat{x}) | \lesssim \hat{x}_n, \end{aligned}$$

hold. Moreover, $\hat{V}/\hat{x}_n$ belongs to $C^{2,\alpha }$ for some $\alpha \in (0,1)$ with $\alpha \leqq p-1$.

### Proof

We start by noticing that the boundary estimate (5.2) translates into

5.10 $$\begin{aligned} \hat{x}_n\lesssim \hat{V}(\hat{x})\lesssim \hat{x}_n \end{aligned}$$

under the change of variables. Indeed, since

$$\begin{aligned} {{\,\textrm{dist}\,}}(x,\partial \Omega )^2 = \inf _{y'\in {\textbf{R}}^{n-1}} \left( |x'-y'|^2 + (x_n-\gamma (y'))^2\right) , \end{aligned}$$

on the one hand, by choosing $y'=x'$, we immediately deduce that

$$\begin{aligned} {{\,\textrm{dist}\,}}(x,\partial \Omega ) \leqq x_n-\gamma (x') = \hat{x}_n. \end{aligned}$$

On the other hand, as the minimizer $y'$ solves the optimality condition $x'-y' = (x_n-\gamma (y'))\nabla '\gamma (y')$, we find

$$\begin{aligned} \hat{x}_n \leqq |x_n-\gamma (y')| + \Vert \nabla '\gamma \Vert _{L^{\infty }} |x'-y'| \leqq \left( 1+\Vert \nabla '\gamma \Vert _{L^{\infty }}\right) {{\,\textrm{dist}\,}}(x,\partial \Omega ). \end{aligned}$$

Thus ${{\,\textrm{dist}\,}}(x,\partial \Omega )$ is comparable to $\hat{x}_n$, which implies (5.10) via (5.2).

We have to show that this estimate remains true for tangential derivatives. Since Lemma 5.1 asserts $\hat{V} \in C^{3,{\alpha }}$, (5.9) follows directly for $k\in \{0,1,2\}$ via Taylor expansion because the homogeneous boundary conditions are invariant under differentiation in tangential direction. For larger values of *k*, we have to transform the elliptic equation (1.3) into a problem on the half-space. In a similar way as we transformed the parabolic equation, we find that

$$\begin{aligned} -\hat{\nabla }\cdot (A\hat{\nabla }(\hat{\eta }\hat{V})) = \hat{\eta }\hat{V}^p + B\cdot \nabla \hat{V} + C\hat{V}, \end{aligned}$$

for some smooth and bounded functions *B* and *C* on ${\textbf{R}}^n_+$ that depend only on the regularity and the shape of the boundary $\partial \Omega$. Differentiating with respect to tangential variables $x_i$ for any $i<n$, we find that

$$\begin{aligned} -\hat{\nabla }\cdot (A\hat{\nabla }(\hat{\eta }\partial _{\hat{x}_i}\hat{V})) = f \end{aligned}$$

with $f \in C^{1,{\alpha }}$, since e.g. Lemma 5.1 shows $\partial _{\hat{x}_i}( \hat{V}^p) = p \hat{V}^p \frac{\partial _{\hat{x}_i} \hat{V}}{\hat{V}}$ to be the product of a $C^{{3},{\alpha }}$ function with a ratio in which the $C^{2,\alpha }$ numerator and $C^{3,\alpha }$ denominator both vanish linearly at the halfspace boundary. On any smooth bounded subdomain of the halfspace containing $\phi (\Omega \cap B_{2r(x_0)})$, Schauder theory (e.g., Chapter 6 in [34]), then implies $\hat{\eta }\partial _{\hat{x}_i}\hat{V} \in C^{3,\alpha }$ so that (5.9) holds for $k=3$. For larger *k*, choosing a sequence $\hat{\eta }_{k-1} > \hat{\eta }_{k}$ of nested cutoffs satisfying the same hypotheses as $\hat{\eta }_3=\hat{\eta }$, and a multi-index $\beta \in \textbf{N}_0^{n-1} \times \{0\}$ consisting of $|\beta |=k-2$ tangential derivatives yields

$$\begin{aligned} -\hat{\nabla }\cdot (A\hat{\nabla }(\hat{\eta }_k \hat{D}^\beta \hat{V})) = f_\beta . \end{aligned}$$

Induction on *k* gives $f_\beta \in C^{1,{\alpha }}$ hence $\hat{\eta }_{k} \hat{D}^\beta \hat{V} \in C^{3,{\alpha }}$ and (5.9) for all *k*; this induction relies on the decay already established for the derivatives of *V* which appear in the *p*-homogeneous nonlinearities, (and the fact that of the $k-1$ derivatives of *V* that contribute elsewhere to $f_\beta$, all but two are in tangential directions).

Finally, the third statement of the lemma follows from Lemma 5.1 and Taylor expansion.

It follows immediately from the preceding lemma that the problem in (5.8) can be further rewritten as

5.11 $$\begin{aligned} \partial _t \hat{H} - \hat{x}_n^{-1-p} \hat{\nabla }\cdot (\hat{x}_n^2 \tilde{A} \hat{\nabla }\hat{H}) = \tilde{F} + \tilde{G} \end{aligned}$$

for some new elliptic $\tilde{A}$, and where $\tilde{G}$ is the sum of $\hat{G}$ and other lower-order terms of the same class. The weak formulation in (5.5) now turns into

5.12 $$\begin{aligned} \begin{aligned}&-\iint _{(0,T)\times {\textbf{R}}^n_+} \hat{H} \partial _t \hat{\varphi }\, \hbox {d}\hat{\mu }_{p+1}\hbox {d}t + \iint _{(0,T)\times {\textbf{R}}^n_+} \hat{\nabla }\hat{\varphi }\cdot \tilde{A} \hat{\nabla }\hat{H}\, \hbox {d} \hat{\mu }_2\hbox {d}t\\&= \iint _{(0,T)\times {\textbf{R}}^n_+} \hat{\varphi }(\hat{F}+\tilde{G})\, \hbox {d} \hat{\mu }_{p+1}\hbox {d}t + \int _{{\textbf{R}}^n_+} \hat{H}_0 \left. \hat{\varphi }\right| _{t=0}\, \hbox {d}\hat{\mu }_{p+1}, \end{aligned} \end{aligned}$$

for any $\hat{\varphi }\in L^2(L^2_2)\cap L^2(\dot{H}^1_2)\cap \dot{H}^1(L^2_{p+1})$ vanishing near the endpoint *T*.

We now prove maximal regularity for the problem in (5.11).

### Lemma 5.4

(Maximal regularity for linearized inhomogeneous halfspace problem) Let $\tilde{F}$ and $\tilde{G}$ be given in $L^2(L^2_{2p})$ and let $\hat{H}\in L^2(L^2_{p+1})\cap L^2(\dot{H}^1_2)$ be a weak solution of (5.11) with zero initial data. Then $\hat{H} \in L^2(\dot{H}^1)\cap L^2(\dot{H}^2_2)\cap \dot{H}^1(L^2_{2p})\cap C^0(L^2_{p+1})$ with

$$\begin{aligned} \begin{aligned}&\Vert \hat{\nabla }\hat{H}\Vert _{L^{\infty }(L^2_{p+1})} + \Vert \partial _t \hat{H}\Vert _{L^2(L^2_{2p})} + \Vert \hat{\nabla }\hat{H}\Vert _{L^2(L^2)} + \Vert \nabla ^2\hat{H}\Vert _{L^2(L^2_2)}\\&\lesssim \Vert \tilde{F}\Vert _{L^2(L^2_{2p})} + \Vert \tilde{G}\Vert _{L^2(L^2_{2p})} + \Vert \hat{\nabla }\hat{H}\Vert _{L^2(L^2_2)} +\Vert \hat{H}\Vert _{L^2(L^2_{2p})} . \end{aligned} \end{aligned}$$

### Proof

In order to simplify the notation, we drop the hats and tildes from here on. Moreover, we set $G=0$. We will here only give formal arguments. The estimates can be derived rigorously by approximating *F* smoothly and using suitable finite difference quotient approximations for the test functions, see, e.g., Chapter 6.3 in [30].

In a first step, we use (an approximation with smooth cut-offs in time of) $\varphi = -\chi _{(0,T)}\partial _k^2\,H$ for some $k\in \{1,\dots ,n-1\}$ as a test function in (5.12), where $\chi _{(0,T)}$ is the characteristic function for the time interval, and we obtain

$$\begin{aligned}&\iint _{(0,\infty )\times {\textbf{R}}^n_+} H\partial _k^2\partial _t (\chi _{(0,T)}H)\, \hbox {d}\mu _{p+1}\hbox {d}t - \iint _{(0,T)\times {\textbf{R}}^n_+} \nabla (\partial _k^2 H)\cdot A \nabla H\, \hbox {d}\mu _2 \hbox {d}t\\&= - \iint _{(0,T)\times {\textbf{R}}^n_+} \partial _k^2 H F\, \hbox {d}\mu _{p+1}\hbox {d}t. \end{aligned}$$

Multiple integrations by parts then yield

5.13 $$\begin{aligned} \begin{aligned}&\frac{1}{2} \int _0^T \frac{\hbox {d}}{\hbox {d}t}\int _{{\textbf{R}}^n_+} (\partial _k H)^2\, \hbox {d}\mu _{p+1}\hbox {d}t + \iint _{(0,T)\times {\textbf{R}}^n_+} \nabla \partial _k H \cdot A\nabla \partial _k H\, \hbox {d}\mu _2 \hbox {d}t \\&= -\iint _{(0,T)\times {\textbf{R}}^n_+} \partial _k^2 H F\, \hbox {d}\mu _{p+1}\hbox {d}t - \iint _{(0,T)\times {\textbf{R}}^n_+} \nabla \partial _k H\cdot (\partial _k A)\nabla H\, \hbox {d}\mu _2\hbox {d}t, \end{aligned} \end{aligned}$$

and invoking the ellipticity of the matrix *A*, the Cauchy–Schwarz inequality and recalling that *H* was assumed to have zero initial data yields

$$\begin{aligned} \Vert \partial _k H\Vert _{L^{\infty }(L^2_{p+1})} + \Vert \nabla \partial _k H\Vert _{L^2(L^2_2)} \lesssim \Vert \partial _k^2 H\Vert _{L^2{(L^2_2)}}^{\frac{1}{2}} \Vert F\Vert _{L^2(L^2_{2p})}^{\frac{1}{2}}+ \Vert \nabla H\Vert _{L^2(L^2_2)}. \end{aligned}$$

Via the elementary estimate $ab \lesssim \varepsilon a^2 + \varepsilon ^{-1}b^2$, we can control the second order term on the right-hand side. We have thus derived the desired control over the second order tangential and mixed derivatives, namely

$$\begin{aligned} \Vert \partial _k H\Vert _{L^{\infty }(L^2_{p+1})} + \Vert \nabla \partial _k H\Vert _{L^2(L^2_2)} \lesssim \Vert F\Vert _{L^2(L^2_{2p})} + \Vert \nabla H\Vert _{L^2(L^2_2)}. \end{aligned}$$

Moreover, an application of Lemma 5.2 provides also the control over the first order tangential derivatives, because $p+1>2$ implies:

$$\begin{aligned} \Vert x_n^{-p} \partial _k H\Vert _{L^2_{2p}} = \Vert \partial _k H\Vert _{L^2} \lesssim \Vert \partial _k H\Vert _{L^2_{p+1}} + \Vert \nabla \partial _k H\Vert _{L^2_2}. \end{aligned}$$

Notice that a replacing the time interval (0, *T*) in (5.13) by $(t,t+\varepsilon )$ shows also the continuity of $\Vert \partial _k H\Vert _{L^2_{p+1}}$ in time.

In order to control the transversal derivatives, it is now enough to focus on the $x_n$ variable, and thus study the one-dimensional problem

$$\begin{aligned} \partial _t H - x_n^{-1-p} \partial _n (x_n^2 A_{nn}\partial _n H) = F, \end{aligned}$$

because all the other terms that appear in (5.11) are now known to belong to $L^2_{2p}$. In order to simplify the notation further, we drop the subscripted *n*’s in the rest of the proof. Furthermore, the problem becomes more accessible if we freeze the diffusivity function *A* at an arbitrary point $x_*$. That is, we study the equation

$$\begin{aligned} \partial _t H - A_* x^{-1-p}\partial _x (x^2 \partial _x H) = F - x^{-1-p}\partial _x (x^2 (A_*-A)\partial _x H), \end{aligned}$$

where we have set $A_*=A(x_*)$. Thanks to the regularity of *A*, it holds that $|A_*-A(x)|\lesssim \delta$ for *x* and $x_*$ in the interval $(0,\delta )$. We should thus localize the problem further by smuggling in a cut-off function $\eta$ satisfying $\eta =1$ in $(0,\delta )$ and vanishing outside of $(0,2\delta )$. This way, we are led to considering

$$\begin{aligned}&\partial _t (\eta H) - A_* x^{-1-p}\partial _x (x^2 \partial _x (\eta H))\\&= \eta F + (A-A_*) x^{-1-p} \partial _x (x^2 \partial _x( \eta H)) -2x^{1-p}\partial _x \eta A\partial _x H\\&\quad -2 x^{-p}\partial _x \eta AH + x^{1-p}\eta \partial _x A\partial _xH - x^{1-p}\partial _x^2\eta A H, \end{aligned}$$

and we write $\tilde{F}$ for the right-hand side for brevity and set $\tilde{H} = \eta H$. Now, if we can show that

5.14 $$\begin{aligned} \Vert \partial _t \tilde{H}\Vert _{L^2_{2p}} +\Vert \partial _x \tilde{H}\Vert _{L^2} + \Vert \partial _x^2 \tilde{H}\Vert _{L^2_2} \lesssim \Vert \tilde{F}\Vert _{L^2_{2p}}, \end{aligned}$$

the statement follows if $\delta$ is sufficiently small, because

$$\begin{aligned} \Vert \tilde{F}\Vert _{L^2_{2p}} \lesssim \Vert F\Vert _{L^2_{2p}} + \delta \Vert \partial _x \tilde{H}\Vert _{L^2} + \delta \Vert \partial _x^2\tilde{H}\Vert _{L^2_2} + \frac{1}{\delta } \Vert \partial _x H\Vert _{L^2_2} +\frac{1}{\delta } \Vert H\Vert _{L^2}, \end{aligned}$$

by the regularity of *A* and the properties of the cut-off function. In view of the interpolation Lemma 5.2, the $L^2$ norm on *H* can be replaced by the $L^2_{2p}$ as in the statement of the lemma.

We have now reduced the multi-dimensional problem with variable coefficients (5.11) to a one-dimension problem with constant coefficient,

$$\begin{aligned} \partial _t \tilde{H} - A_* x^{-1-p}\partial _x (x^2 \partial _x \tilde{H}) =\tilde{F}. \end{aligned}$$

We have to do one more transformation in order to arrive at a problem that is better behaved. Indeed, if we change variables $\check{x}= x^{p+1}$, $\check{t} = A_* (p+1)^2t$, $\check{H}(\check{t},\check{x}) = \tilde{H}(t,x)$ and $\check{ F}(\check{t},\check{x}) = \tilde{F}(t,x)$ the above equation turns into

$$\begin{aligned} \partial _{\check{t}} \check{H} - \check{x}^{-\sigma } \partial _{\check{x}}(\check{x}^{\sigma +1}\partial _{\check{x}} \check{H}) = \check{F}, \end{aligned}$$

where $\sigma = \frac{1}{p+1}$. This is precisely the linear version of the parabolic equation that characterizes the porous medium dynamics in a neighborhood of the Barenblatt solution as studied earlier in [38, 40, 48]. It is well-understood: Calderón–Zygmund and Muckenhoupt theory is available and provides estimates

$$\begin{aligned} \Vert \partial _{\check{t}}\check{H}\Vert _{L^2_q} + \Vert \partial _{\check{x}}\check{H}\Vert _{L^2_{q}} + \Vert \partial _{\check{x}}^2 \check{H}\Vert _{L^2_{q+2}} \lesssim \Vert \check{F}\Vert _{L^2_q}, \end{aligned}$$

for any $q\in (-1,2(\sigma +1)-1)$, see the proofs of Proposition 3.23 in [38] or Proposition 4.23 in [48]. Our choice is $q = \frac{p}{p+1}$, which is equivalent to (5.14).

We summarize our findings as follows:

### Proposition 5.5

(Linear inhomogenous a priori estimates) Let *h* be a weak solution to the linear equation (5.5) with zero initial datum and $f\in L^2(L^2_{2p}){\cap L^1(L^2_{p+1}})$. Then for all $T>0$, the following holds:

5.15 $$\begin{aligned} \begin{aligned}&\Vert \nabla h\Vert _{L^{\infty }((0,T);L^2_{p+1})}+ \Vert \partial _t h\Vert _{L^2((0,T);L^2_{2p})} + \Vert \nabla h\Vert _{L^2((0,T);L^2)} + \Vert \nabla ^2 h\Vert _{L^2((0,T);L^2_{2})}\\&\lesssim \Vert f\Vert _{L^2((0,T);L^2_{2p})} + {\Vert h\Vert _{L^2((0,T);L^2_{2p})}}. \end{aligned} \end{aligned}$$

### Proof

Since $\Omega \subset {\textbf{R}}^n$ is bounded, its boundary can be covered by finitely many open balls, sufficiently small that within each of them, the boundary can be expressed as a graph over any of its tangent planes. The complement of these open sets in $\Omega$ can be covered by one additional open set compactly contained in the interior of $\Omega$. Choosing a partition of unity subordinate to this covering, we flatten the boundary in each of the covering balls and apply Lemma 5.4. The analogous estimates in the interior of $\Omega$ follow from standard parabolic estimates and the boundedness of $\log V$. Combining these estimates in the original variables using the partition of unity, the proposition follows from a linear analog of Lemma 4.1.

This maximal regularity result can be easily combined with the energy estimate for the nonlinear problem.

### Lemma 5.6

(Nonlinear smoothing 1) Let *h* be a solution to the nonlinear equation (2.1) with initial datum $h_0\in L^2_{p+1}$ and assume that $\Vert h\Vert _{L^{\infty }}\leqq \varepsilon$ for some $\varepsilon >0$ small enough. Then, for any $0<\tau<1<T$ there exists $C=C(\tau ,T,n,p,V)$ such that

5.16 $$\begin{aligned} \Vert \partial _t h\Vert _{L^2((\tau ,T);L^2_{2p}))} + \Vert \nabla h\Vert _{L^2((\tau ,T);L^2(L^2))} + \Vert \nabla ^2 h\Vert _{L^2((\tau ,T);L^2_{2}))} \leqq C \Vert h_0\Vert _{L^2_{p+1}}. \end{aligned}$$

### Proof

We denote by $\zeta$ a smooth cut-off function that is 1 in the time interval $[\tau , T]$ and zero in $[0,\tau /2]$. We localize the evolution (2.1) with the help of this function

5.17 $$\begin{aligned} \partial _t(\zeta h) - V^{-1-p}\nabla \cdot (V^2 \nabla (\zeta h)) = (p-1)\zeta h + h \partial _t \zeta + \zeta N(h), \end{aligned}$$

and apply the maximal regularity estimate from Proposition 5.5 to the effect that

$$\begin{aligned}&\Vert \partial _t(\zeta h)\Vert _{L^2(L^2_{2p})} + \Vert \nabla (\zeta h)\Vert _{L^2(L^2)} + \Vert \nabla ^2 (\zeta h)\Vert _{L^2(L^2_2)}\\&\lesssim {\Vert \zeta h\Vert _{L^2(L^2_{2p})} + \Vert h \partial _t \zeta \Vert _{L^2(L^2_{2p})} +\Vert \zeta N(h)\Vert _{L^2(L^2_{2p})}}. \end{aligned}$$

Thanks to the particular structure of the nonlinearity (2.2), the third term on the right hand side can be estimated by

$$\begin{aligned} {\Vert \zeta N(h)\Vert _{L^2(L^2_{2p})} \lesssim \Vert h \partial _t \zeta \Vert _{L^2(L^2_{2p})} + \Vert \zeta h\Vert _{L^2(L^2_{2p})} + \varepsilon \Vert \partial _t(\zeta h)\Vert _{L^2(L^2_{2p})}.} \end{aligned}$$

Using the fact that $L^2_{p+1}$ embeds continuously into $L^2_{2p}$ by the virtue of (5.2), the above estimates combine with the pointwise bounds from Lemma 4.1 to give

$$\begin{aligned}&\Vert \partial _t(\zeta h)\Vert _{L^2(L^2_{2p})} + \Vert \nabla (\zeta h)\Vert _{L^2(L^2)} + \Vert \nabla ^2 (\zeta h)\Vert _{L^2(L^2_2)} \\&\leqq C\left( \varepsilon \Vert \partial _t(\zeta h)\Vert _{L^2(L^2_{2p})} + \Vert h_0\Vert _{L^2_{p+1}}\right) . \end{aligned}$$

Choosing $\varepsilon$ small enough and invoking the properties of the cut-off function yields the statement of the lemma.

Before turning to higher-order derivatives, we use integration by parts to establish a class of interpolation inequalities which will allow us to control the effects of the nonlinearity.

### Lemma 5.7

(Interpolation) Let $\psi \in C^{\infty }_c({\textbf{R}}^n)$ be given, $q\geqq 2$ and $k,\ell \in \textbf{N}$ with $k>\ell$. Then it holds

$$\begin{aligned} \Vert \psi |D^{\ell } h|^{\frac{k}{\ell }}\Vert _{L^q} \lesssim \Vert h\Vert _{L^{\infty }}^{\frac{k-\ell }{\ell }} \sum _{m=0}^{k-1} \Vert D^m\psi D^{k-m}h\Vert _{L^q}. \end{aligned}$$

### Proof

To keep the notation as simple as possible, we perform a rather symbolic calculation. That is, we write $\partial ^m$ for some partial derivative of *m*th order, $\partial ^m = \partial _x^{\alpha }$ with $|\alpha |=m$. In our argument, the precise value of $\alpha$ is not of importance.

We start with an integration by parts to notice that

$$\begin{aligned}&\int |\psi |^q |\partial ^{\ell } h|^{\frac{kq}{\ell }}\, \hbox {d}x\\&\quad = \int |\psi |^q \partial ^{\ell } h \partial ^{\ell } h |\partial ^{\ell } h|^{\frac{kq-2\ell }{\ell }}\, \hbox {d}x\\&\quad \lesssim \int |\psi |^q |\partial ^{\ell -1}h||\partial ^{\ell +1}h||\partial ^{\ell }h|^{\frac{kq-2\ell }{\ell }}\, \hbox {d}x + \int |\psi |^{q-1} |\partial \psi | |\partial ^{\ell -1}h| |\partial ^{\ell }h|^{\frac{kq-\ell }{\ell }}\, \hbox {d}x\\&\quad \lesssim \left( \int |\psi |^q |\partial ^{\ell -1} h|^{\frac{kq}{\ell -1}}\, \hbox {d}x\right) ^{\frac{\ell -1}{kq}}\left( \int |\psi |^q |\partial ^{\ell +1} h|^{\frac{kq}{\ell +1}}\, \hbox {d}x\right) ^{\frac{\ell +1}{kq}} \left( \int |\psi |^q |\partial ^{\ell }h|^{\frac{kq}{\ell }}\, \hbox {d}x\right) ^{\frac{kq-2\ell }{kq}}\\&\qquad + \left( \int |\psi |^q |\partial ^{\ell -1} h|^{\frac{kq}{\ell -1}}\, \hbox {d}x\right) ^{\frac{\ell -1}{kq}}\left( \int |\psi |^q |\partial ^{\ell } h|^{\frac{kq}{\ell }}\, \hbox {d}x\right) ^{\frac{kq-k-\ell +1}{kq}} \left( \int |\partial \psi |^q |\partial ^{\ell }h|^{\frac{(k-1)q}{\ell }}\, \hbox {d}x\right) ^{\frac{1}{q}}, \end{aligned}$$

where the last estimates follow from Hölder’s inequality. Here, we employ the convention that

$$\begin{aligned} \left( \int |\psi |^q |\partial ^{\ell -1} h|^{\frac{kq}{\ell -1}}\, \hbox {d}x\right) ^{\frac{\ell -1}{kq}} = \Vert h\Vert _{L^{\infty }}\quad \text{ for } \ell =1. \end{aligned}$$

Applying Young’s inequality in the form $ab \lesssim \varepsilon a^{\frac{1}{\theta }} + C_{\varepsilon ,\theta } b^{\frac{1}{1-\theta }}$ for some arbitrarily small $\varepsilon$ and $\theta \in (0,1)$ to the right hand side yields

$$\begin{aligned}\begin{aligned} \int |\psi |^q |\partial ^{\ell } h|^{\frac{kq}{\ell }}\,\hbox {d}x&\lesssim \left( \int |\psi |^q |\partial ^{\ell -1} h|^{\frac{kq}{\ell -1}}\, \hbox {d}x\right) ^{\frac{\ell -1}{2\ell }}\left( \int |\psi |^q |\partial ^{\ell +1}h|^{\frac{kq}{\ell +1}}\, \hbox {d}x\right) ^{\frac{\ell +1}{2\ell }}\\&\quad + \left( \int |\psi |^q |\partial ^{\ell -1} h|^{\frac{kq}{\ell -1}}\, \hbox {d}x\right) ^{\frac{\ell -1}{k+\ell -1}}\left( \int |\partial \psi |^q |\partial ^{\ell }h|^{\frac{(k-1)q}{\ell }}\, \hbox {d}x\right) ^{\frac{k}{k+\ell -1}}.\end{aligned} \end{aligned}$$

It remains to apply an iteration procedure. For this purpose, we set, for $k\geqq \ell +m$,

$$\begin{aligned} A(k,\ell ,m)&:= \left( \int |D^m\psi |^q |D^{\ell }h |^{\frac{(k-m)q}{\ell }}\, \hbox {d}x\right) ^{\frac{\ell }{(k-m)q}},\\ B(\ell ,m)&: = A(\ell +m,\ell ,m),\\ C(k,\ell )&: = A(k,\ell ,0) \end{aligned}$$

and notice that $A_0:= A(k,0,m) = \Vert h\Vert _{L^{\infty }}$. Upon rescaling *h*, we may assume from here on that $A_0=1$. (We may always assume $A_0 \ne 0$ as otherwise the lemma is vacuously true.) With this notation, the previous estimate becomes

5.18 $$\begin{aligned} C(k,\ell ) \lesssim C(k,\ell -1)^{\frac{1}{2}}C(k,\ell +1)^{\frac{1}{2}} + C(k,\ell -1)^{\frac{\ell }{k+\ell -1}} A(k,\ell ,1)^{\frac{k-1}{k+\ell -1}}, \end{aligned}$$

and the statement of the lemma can be rephrased as

5.19 $$\begin{aligned} C(k,\ell )^{\frac{k}{\ell }}\lesssim \sum _{m=0}^{k-1} B(k-m,m), \end{aligned}$$

for every *k* and $1\leqq \ell \leqq k-1$.

The proof will be a double-induction on $(k,\ell )$. We start by noticing that for $k=2$ and $\ell =1$, our objective (5.19) is nothing but the estimate (5.18) just proven. Suppose $k\geqq 3$ is fixed and that (5.19) holds true for $k-1$ and any $\ell \leqq k-2$. Our goal is to show (5.19) for fixed *k* and all $1\leqq \ell \leqq k-1$.

We first need some auxiliary inequalities. Note for $\varphi = D\psi$, it holds that

$$\begin{aligned} A(k,\ell ,m)&= \left( \int |D^{m-1}\varphi |^q |D^{\ell }h|^{\frac{((k-1)- (m-1))q}{\ell }} \, \hbox {d}x\right) ^{\frac{\ell }{((k-1)- (m-1))q}} \\ {}&=: \tilde{A}(k-1,\ell ,m-1) \end{aligned}$$

and since the estimate in (5.18) is independent of the choice $\psi$, the inductive hypothesis (5.19) allows us to estimate

$$\begin{aligned} \tilde{A}(k-1,\ell ,0)^{\frac{k-1}{\ell }} =: \tilde{C}(k-1,\ell )^{\frac{k-1}{\ell }}&\lesssim \sum _{m=0}^{k-1-1} \tilde{B}(k-1-m,m) \\ {}&:= \sum _{m=0}^{k-1-1} \tilde{A}(k-1,k-1-m,m) \end{aligned}$$

or

$$\begin{aligned} A(k,\ell ,1))^{\frac{k-1}{\ell }} \lesssim \sum _{m=1}^{k-1} B(k-m,m), \end{aligned}$$

for any $\ell \leqq k-2$. Plugging this bound into (5.18) gives

5.20 $$\begin{aligned} \begin{aligned} C(k,\ell )&\lesssim C(k,\ell -1)^{\frac{1}{2}} C(k,\ell +1)^{\frac{1}{2}} \\&\quad + C(k,\ell -1)^{\frac{\ell }{k+\ell -1}} \left( {\sum _{m=1}^{k-1} B(k-m,m)}\right) ^{\frac{\ell }{k+\ell -1}}. \end{aligned} \end{aligned}$$

We claim that this estimate implies

5.21 $$\begin{aligned} C(k,\ell )^{\frac{k}{\ell }} \lesssim C(k,\ell +1)^{\frac{k}{\ell +1}} + \sum _{m=1}^{k-1} B(k-m,m) \end{aligned}$$

for any $1\leqq \ell \leqq k-1$. Indeed, the case $\ell =1$ follows directly from (5.20) because $C(k,0)=A_0=1$. The general case follows by induction: We suppose that (5.21) is proved for $1,2,\dots ,\ell -1$ and we aim at establishing it for $\ell$. For this purpose, we use Young’s inequality in (5.20) to the effect that

$$\begin{aligned} C(k,\ell )^{\frac{k}{\ell }} \lesssim \varepsilon C(k,\ell -1)^{\frac{k}{\ell -1}} + C(k,\ell +1)^{\frac{k}{\ell +1}} + \sum _{m=1}^{k-1}B(k-m,m), \end{aligned}$$

for some arbitrary $\varepsilon$. Invoking the hypothesis that (5.21) holds true for $\ell -1$, we then deduce

$$\begin{aligned} C(k,\ell )^{\frac{k}{\ell }} \lesssim \varepsilon C(k,\ell )^{\frac{k}{\ell }} + C(k,\ell +1)^{\frac{k}{\ell +1}} + \sum _{m=1}^{k-1}B(k-m,m), \end{aligned}$$

which gives (5.21) for $\ell$ if $\varepsilon$ is chosen small enough.

It remains to iterate (5.21) to find

$$\begin{aligned} C(k,\ell )^{\frac{k}{\ell }} \lesssim C(k,k) + \sum _{m=1}^{k-1} B(k-m,m) = \sum _{m=0}^{k-1} B(k-m,m), \end{aligned}$$

which is what we aimed to prove, cf. (5.19).

We will now perform an intermediate step towards higher-order regularity estimates by lifting the norms on the left-hand side in (5.16) to the next order in time. Higher-order time derivatives will be considered subsequently simultaneously with suitable higher-order spatial derivatives. The intermediate step that we take in the following lemma is necessary in order to control lower-order error terms that appear later as a result of a transformation of the equation close to the boundary; see (5.22) below.

### Lemma 5.8

(Nonlinear smoothing 2) Let *h* be a solution to the equation (2.1) with initial datum $h_0\in L^2_{p+1}$ and assume that $\Vert h\Vert _{L^{\infty }}\leqq \varepsilon$ for some $\varepsilon >0$. Then if $\varepsilon$ is small enough and $0<\tau<1<T$, it holds that

$$\begin{aligned} \Vert \partial _t^2 h\Vert _{L^2((\tau ,T);L^2_{2p}))} + \Vert \nabla \partial _t h\Vert _{L^2((\tau ,T);L^2(L^2))} + \Vert \nabla ^2\partial _t h\Vert _{L^2((\tau ,T);L^2_{2}))}\lesssim \Vert h_0\Vert _{L^2_{p+1}}. \end{aligned}$$

### Proof

Regularity in time was proved already by Jin and Xiong, see (5.1) above. In order to get control over the mixed derivatives, we proceed carefully by considering finite difference quotients $d_t^s h (t) = s^{-1}(h(t+s)-h(s))$. We consider the same cut-off function in time as in the proof of Lemma 5.6. Then localizing the nonlinear equation and “differentiating” (5.17) with respect to time, we obtain

$$\begin{aligned}&\partial _t d_t^s (\zeta h) -V^{1-p}\nabla \cdot (V^2\nabla d_t^s(\zeta h)) \\&= (p-1)d_t^s (\zeta h) + d_t^s h \partial _t \zeta + hd_t^s \partial _t \zeta +\zeta d_t^s N(h) + N(h) d_t^s \zeta . \end{aligned}$$

Regarding the nonlinear terms, we notice that

$$\begin{aligned} |N(h)| \lesssim |h|^2 + |h||\partial _t h| \end{aligned}$$

and

$$\begin{aligned} |d_t^s {N}(h)| \lesssim |h||d_t^s h| + |d_t^sh||\partial _t h| + |h||\partial _t d_t^s h|. \end{aligned}$$

Therefore, using $|h|\leqq \varepsilon \leqq 1$ and making use of the maximal regularity estimate for the linear problem, Proposition 5.5, we find that

$$\begin{aligned}&\Vert \partial _t d_t^s(\zeta h)\Vert _{L^2(L^2_{2p})} + \Vert \nabla d_t^s(\zeta h)\Vert _{L^2(L^2)} + \Vert \nabla ^2 d_t^s (\zeta h)\Vert _{L^2(L^2_2)}\\&\lesssim \Vert \chi _{{{\,\textrm{spt}\,}}\zeta } h\Vert _{L^2(L^2_{2p})} + \Vert \chi _{{{\,\textrm{spt}\,}}\zeta } d_t^s h\Vert _{L^2(L^2_{2p})}\\&\quad + \Vert \zeta d_t^sh\partial _t h\Vert _{L^2(L^2_{2p})} + \varepsilon \Vert d_t^s\partial _t(\zeta h)\Vert _{L^2(L^2_{2p})} . \end{aligned}$$

If $\varepsilon$ is sufficiently small, the last term on the right-hand side can be absorbed into the left-hand side. Moreover, the (remaining) expressions on the right-hand side are bounded uniformly in *s* by the virtue of Jin and Xiong’s regularity statement (5.1). We may thus pass to the limit $s\rightarrow 0$ and find

$$\begin{aligned}&\Vert \partial _t^2(\zeta h)\Vert _{L^2(L^2_{2p})} + \Vert \nabla \partial _t (\zeta h)\Vert _{L^2(L^2)} + \Vert \nabla ^2 \partial _t (\zeta h)\Vert _{L^2(L^2_2)}\\&\lesssim \Vert \chi _{{{\,\textrm{spt}\,}}\zeta } h\Vert _{L^2(L^2_{2p})} + \Vert \chi _{{{\,\textrm{spt}\,}}\zeta } \partial _t h\Vert _{L^2(L^2_{2p})}+ \Vert \zeta (\partial _t h)^2\Vert _{L^2(L^2_{2p})} . \end{aligned}$$

Now, applying the interpolation Lemma 5.7 in the form

$$\begin{aligned} \Vert \zeta |\partial _t h|^2 \Vert _{L^2(L^2_{2p})} \lesssim \Vert h\Vert _{L^{\infty }} \Vert \zeta \partial _t^2 h\Vert _{L^2(L^2_{2p})} + \Vert h\Vert _{L^{\infty }}\Vert \partial _t\zeta \partial _t h\Vert _{L^2(L^2_{2p})}, \end{aligned}$$

and using again that $|h|\leqq \varepsilon \leqq 1$ leads us to the estimate

$$\begin{aligned}&\Vert \partial _t^2(\zeta h)\Vert _{L^2(L^2_{2p})} + \Vert \nabla \partial _t(\zeta h)\Vert _{L^2(L^2)} + \Vert \nabla ^2 \partial _t (\zeta h)\Vert _{L^2(L^2_2)}\\&\lesssim \Vert \chi _{{{\,\textrm{spt}\,}}\zeta } h\Vert _{L^2(L^2_{2p})} + \Vert \chi _{{{\,\textrm{spt}\,}}\zeta } \partial _t h\Vert _{L^2(L^2_{2p})} , \end{aligned}$$

provided that $\varepsilon$ is sufficiently small. Since $\mu _{2p}\lesssim \mu _{p+1}$ by the virtue of Lemma 5.1, we can now apply Lemmas 4.1 and 5.6 and deduce the statement of the lemma by the properties of the cut-off function.

Similarly to the derivation of the maximal regularity estimate in Proposition 5.5, the derivation of higher-order regularity estimates requires attention only in a neighborhood of the boundary. Indeed, in the interior the equation is parabolic with smooth coefficients, and thus, higher-order estimates in the interior just follow by standard iterative arguments based on the maximal regularity estimate from Proposition 5.5. As before, we shall thus focus on the boundary from here on. We choose essentially the same notation as in the proof of Proposition 5.5 and we fix $x_0\in \partial \Omega$ arbitrarily and let $\eta$ denote a cut-off function on ${\textbf{R}}^n$ interpolating smoothly between $\eta =1$ in $B_r(x_0)$ and $\eta = 0$ outside of $B_{2r}(x_0)$. Moreover, as in the proofs of Lemmas 5.6 and 5.8, we have to introduce a cut-off function $\zeta$ defined on $[0,\infty )$ that satisfies $\zeta =0$ in $[0,\tau /2]$ and $\zeta =1$ in $[\tau ,T]$ for some $0<\tau<1<T$. Treating the nonlinearity $N(h(x))=:f(x)$ as an inhomogeneity and smuggling $\zeta \eta$ into the equation, we find that $H = \zeta \eta h$ satisfies

5.22 $$\begin{aligned} \partial _t H - V^{-1-p}\nabla \cdot (V^2\nabla H) = (p-1)H + F+G, \end{aligned}$$

where

$$\begin{aligned} F = \zeta \eta f,\quad G = -2V^{1-p}\zeta \nabla \eta \cdot \nabla h - V^{1-p}\zeta h \Delta \eta - 2\zeta V^{-p} h \nabla V \cdot \nabla \eta + \zeta ' \eta h. \end{aligned}$$

We now apply the same diffeomorphism $\phi$ that we used in order to transform the elliptic problem (5.7) into the half-space problem (5.11), and arrive at

5.23 $$\begin{aligned} \partial _t \hat{H} - \hat{V}^{-1-p}\hat{\nabla }\cdot (\hat{V}^2 A\hat{\nabla }\hat{H}) = (p-1)\hat{H} +\hat{F} + \hat{G}. \end{aligned}$$

We will now derive control on higher-order derivatives for equation (5.23). As before, we interpret the weighted Lebesgue norms with respect to the simpler weight $\hat{x}_n$ and we consider $L^r(L^2_{q}) = L^r((0,T);L^2(\hat{\mu }_q))$ with measure $d\hat{\mu }_q = \hat{x}_n^q\, d\hat{x}$ and typically $r=2$. Moreover, we write $z=(t,\hat{x}')$ for the time and flattened tangential variables, whereas $\hat{\nabla }$ denotes the full spatial gradient (tangential and normal) in flattened coordinates.

### Lemma 5.9

(Tangential smoothing by the linear inhomogeneous evolution) Let $\hat{H}$ be a solution to the transformed equation (5.23). For any $k\in \textbf{N}$ and $\alpha '\in \textbf{N}_0^{n}$ with $|\alpha '|=k$, it holds that

5.24 $$\begin{aligned} \begin{aligned}&\Vert D_z^k\partial _t \hat{H}\Vert _{L^2(L^2_{2p})} + \Vert D_z^k \hat{\nabla }\hat{H}\Vert _{L^2(L^2)} + \Vert D_z^k \hat{\nabla }^2 \hat{H}\Vert _{L^2(L^2_{2})}\\&\lesssim \sum _{\ell =0}^k \Vert D_z^{\ell }\hat{F}\Vert _{L^2(L^2_{2p})} + \sum _{\ell =0}^k \Vert D_z^{\ell } \hat{G}\Vert _{L^2(L^2_{2p})}. \end{aligned} \end{aligned}$$

### Proof

As $\hat{G}$ can be considered as an inhomogeneity, we can set $\hat{G}=0$ for notational convenience.

We can proceed as in the proof of Proposition 5.5 and show that (5.15) holds true on the half-space, that is, we have

5.25 $$\begin{aligned} \Vert \partial _t \hat{H}\Vert _{L^2(L^2_{2p})} + \Vert \hat{\nabla }\hat{H}\Vert _{L^2(L^2)} + \Vert \hat{\nabla }^2 \hat{H}\Vert _{L^2(L^2_{2})}\lesssim \Vert \hat{F}\Vert _{L^2(L^2_{2p})}. \end{aligned}$$

Note that the implicit constant in this estimate might be time-dependent and blow up for infinite times. Now, we differentiate (5.23) in time and tangential direction. For $m\in \textbf{N}_0$ and $\alpha '\in \textbf{N}_0^{n-1}$, it holds that

$$\begin{aligned}&\partial _t \partial _t^m\partial _{\hat{x}'}^{\alpha '} \hat{H} - \hat{V}^{-1-p}\hat{\nabla }\cdot ( V^2 A\hat{\nabla }\partial _t^m\partial _{\hat{x}'}^{\alpha '} \hat{H}) \\&= (p-1)\partial _t^m\partial _{\hat{x}'}^{\alpha '} \hat{H} +\partial _t^m\partial _{\hat{x}'}^{\alpha '} \hat{F}\\&\qquad + \hat{x}_n^{1-p} \sum _{\begin{array}{c} 1\leqq |\beta |\leqq |\alpha '|+1 \\ \beta _n\leqq 2 \end{array}} a_{\beta } \partial _t^m\partial _{\hat{x}}^{\beta } \hat{H} + \hat{x}_n^{-p} \sum _{\begin{array}{c} 1\leqq |\beta |\leqq |\alpha '|\\ \beta _n\leqq 1 \end{array}} b_{\beta } \partial _t^m\partial _{\hat{x}}^{\beta }\hat{H} , \end{aligned}$$

for some continuous and bounded functions $a_{\beta }, b_{\beta }$ on ${\textbf{R}}$. We can now apply the maximal regularity estimate (5.25) and find (5.24) via iteration and thanks to the fact that $\hat{x}_n\lesssim 1$ in the support of $\hat{H}$.

Now, we translate the above estimate to the nonlinear setting. That is, we consider $f=f_0+f_1$, with

$$\begin{aligned} f_0 = (1+h)^p - 1 - p h,\quad f_1 = \left( (1+ h)^{p-1}-1 \right) \partial _t h, \end{aligned}$$

and we write $\hat{F}_i(t,\hat{x}) = \zeta (t) \hat{\eta }(\hat{x}) \hat{f}_i (\hat{x}) = \zeta (t)\eta (x) f_i(x)$ for the transformed and truncated quantities. Moreover, we write $\hat{h}(t,\hat{x}) = h(t,x)$. The nonlinearities are bounded as follows.

### Lemma 5.10

(Spacetime localized boundary estimates for the nonlinearity)

Suppose that $\Vert \hat{h}\Vert _{L^{\infty }}\leqq \varepsilon$ for some $\varepsilon \ll 1$. Then, for any $k\in \textbf{N}_{0}$ there exists a constant $\nu \in (0,1)$ such that

$$\begin{aligned} \begin{aligned} \Vert D_z^k \hat{F}_i\Vert _{L^2(L^2_{2p })} \lesssim \varepsilon ^{\nu } \sum _{m=0}^{k} \Vert D_z^{k-m}(\zeta \hat{\eta }) D_z^{m+i} \hat{h}\Vert _{L^2(L^2_{2p})} \qquad {\forall i \in \{0,1\}}. \end{aligned} \end{aligned}$$

### Proof

We drop the hats for notational convenience. We start considering the estimate for $$ F_0$$, and notice that

$$\begin{aligned} \partial _{z}^{\alpha } F_0 = \sum _{\beta \leqq \alpha } {\alpha \atopwithdelims ()\beta } \partial _{z}^{\alpha - \beta } \psi \, \partial _{z}^{\beta } f_0, \end{aligned}$$

by the multi-dimensional Leibniz rule, where we have set $\psi = \zeta \eta$. We inspect the nonlinearity and find by Young’s inequality and an iterative argument that

$$\begin{aligned} | \partial ^{\beta }_{z} f_0| \lesssim |h| | \partial _{z}^{\beta } h| + \sum _{\begin{array}{c} \gamma \leqq \beta \\ 1\leqq |\gamma |\leqq |\beta |-1 \end{array}} | \partial _{z}^{\gamma } h |^{\frac{|\beta |}{|\gamma |}}, \end{aligned}$$

provided that $\varepsilon$ is sufficiently small and $|\beta |\geqq 1$. Therefore, summing over any multi-indices $\alpha$ with $|\alpha |=k$ and integrating in space and time, we have that

$$\begin{aligned} \Vert D_z^k F_0\Vert _{L^2(L^2_{2p })}&\lesssim \Vert h\Vert _{L^{\infty }} \Vert \psi D_z^k h\Vert _{L^2(L^2_{2p})} + \sum _{\ell =1}^{k-1} \Vert \psi |D_z^{\ell } h |^{\frac{k}{\ell }}\Vert _{L^2(L^2_{2p})} \\ {}&\qquad + \sum _{{m=0}}^{k-1} \Vert h\Vert _{L^{\infty }} \Vert D_z^{k-m} \psi D_z^m h\Vert _{L^2(L^2_{2p})} \\ {}&\qquad + \sum _{{m=2}}^{k-1} \sum _{\ell =1}^{{m-1}} \Vert D_z^{k-m}\psi | D_z^{\ell } h |^{\frac{m}{\ell }}\Vert _{L^2(L^2_{2p})}. \end{aligned}$$

Let’s discuss the right-hand side term by term. The first term is exactly of the kind we are looking for. For the second one, we apply Lemma 5.7 and find,

$$\begin{aligned} \sum _{\ell =1}^{k-1} \Vert \psi |D_z^{\ell } h |^{\frac{k}{\ell }}\Vert _{L^2(L^2_{2p})} \lesssim \varepsilon ^{\nu } \sum _{m=0}^{k-1} \Vert D_z^m\psi D_z^{k-m} h\Vert _{L^2(L^2_{2p})}, \end{aligned}$$

for some $\nu >0$. The remaining terms are bounded by the same quantity.

The treatment of $f_1$ is similar. This time, derivatives of the nonlinearity are bounded as follows,

$$\begin{aligned} |D_z^{\ell } f_1|&\lesssim |h| |D_z^{\ell }\partial _t h| + \sum _{m=0}^{\ell -1} \sum _{n=1}^{\ell -m} |D_z^n h|^{\frac{\ell -m}{n}} |D_z^{m}\partial _t h|, \end{aligned}$$

as can be observed by Young’s inequality and an iterative argument. With the help of the Leibniz rule, we thus obtain

$$\begin{aligned} \Vert D_z^k F_1\Vert _{L^2(L^2_{2p})}&\lesssim \Vert h\Vert _{L^{\infty }} \Vert \psi D_z^k\partial _t h\Vert _{L^2(L^2_{2p})} +\sum _{\ell =0}^{k-1} \Vert h\Vert _{L^{\infty }} \Vert D^{k-\ell }_z \psi D_z^{\ell } \partial _t h\Vert _{L^2(L^2_{2p})} \\ {}&\qquad + \sum _{m=0}^{k-1} \sum _{n=1}^{k-m} \Vert \psi |D_z^n h|^{\frac{k-m}{n}} D_z^{m} \partial _t h\Vert _{L^2(L^2_{2p})} \\ {}&\qquad +\sum _{\ell =0}^{k-1} \sum _{m=0}^{ \ell -1} \sum _{n=1}^{ \ell -m} \Vert D_z^{k-\ell }\psi |D^nh|^{\frac{\ell -m}{n}} D^m_z \partial _t h\Vert _{L^2(L^2_{2p})}, \end{aligned}$$

and absorbing $\partial _t$ into $D_z$ and an application of the Hölder inequality furthermore yields

$$\begin{aligned}&\Vert D_z^k F_1\Vert _{L^2(L^2_{2p})} \\&\quad \lesssim \Vert h\Vert _{L^{\infty }}\left( \Vert \psi D_z^{k+1}h\Vert _{L^2(L^2_{2p})} +\sum _{\ell =1}^{k-1} \Vert D^{k-\ell }_z \psi D_z^{\ell +1} h\Vert _{L^2(L^2_{2p}) }\right) \\&\qquad + \sum _{m=0}^{k-1} \sum _{n=1}^{k-m} \Vert \psi |D_z^{n} h|^{\frac{k+1}{n}}\Vert _{L^2(L^2_{2p})}^{\frac{k-m}{k+1}}\Vert \psi |D_z^{m+1} h|^{\frac{k+1}{m+1}}\Vert _{L^2(L^2_{2p})}^{\frac{m+1}{k+1}}\\&\qquad +\sum _{\ell =0}^{k-1} \sum _{m=0}^{ \ell -1} \sum _{n=1}^{ \ell -m} \Vert D_z^{k-\ell }\psi |D_z^n h|^{\frac{\ell +1}{n}}\Vert _{L^2(L^2_{2p})}^{\frac{\ell -m}{\ell +1}} \Vert D_z^{k-\ell }\psi |D_z^{m+1}h|^{\frac{\ell +1}{m+1}}\Vert _{L^2(L^2_{2p})}^{\frac{m+1}{\ell +1}}. \end{aligned}$$

We now invoke Lemma 5.7 to the effect that

$$\begin{aligned} \Vert D_z^{k} F_1\Vert _{L^2(L^2_{2p})} \lesssim \varepsilon ^{\nu } \sum _{m=0}^k \Vert D_z^m \psi D_z^{k+1-m} h\Vert _{L^2(L^2_{2p})}, \end{aligned}$$

for some $$\nu >0$$.

With these preparations, we are in the position to extend the $L^2$ estimates from Lemmas 4.1, 5.6, and 5.8 to *z*-derivatives of any order where $z=(t,\hat{x}')$ denotes the tangential and time variables and $\hat{\nabla }$ denotes the full spatial gradient (tangential and normal) in flattened coordinates. For our purposes, it is enough to bound the unweighted terms in these estimates.

### Proposition 5.11

(Tangential nonlinear smoothing in Hilbert norms) Let $\hat{H}$ be the solution to the transformed equation (5.23) and suppose that $\Vert \hat{H}\Vert _{L^{\infty }}\leqq \varepsilon$ for some $\varepsilon$ small enough. Let $0<\tau<1<T$ be given. Then, for any $k\in \textbf{N}_0$, it holds that

$$\begin{aligned} \Vert D_z^k \hat{\nabla }\hat{H}\Vert _{L^2(L^2)} + \Vert D_z^k\hat{\nabla }^2 \hat{H}\Vert _{L^2(L^2_2)}\lesssim \Vert h_0\Vert _{L^2_{p+1}}. \end{aligned}$$

### Proof

We start by noting that the estimates from Lemmas 4.1, 5.6 and 5.8 easily translate into the localized setting, so that

5.26 $$\begin{aligned} \begin{aligned}&\Vert \hat{H}\Vert _{L^{\infty }((0,T); L^2_{p+1})} + \Vert \partial _t \hat{H}\Vert _{L^2((\tau ,T);L^2_{2p})}+ \Vert \partial _t^2 \hat{H}\Vert _{L^2((\tau ,T);L^2_{2p})}\\&\quad + \Vert \hat{\nabla }\hat{H}\Vert _{L^2((\tau ,T);L^2)} + \Vert \hat{\nabla }\partial _t \hat{H}\Vert _{L^2((\tau ,T);L^2)} + \Vert \hat{\nabla }^2 \hat{H}\Vert _{L^2((\tau ,T);L^2_2)}\\&\quad + \Vert \hat{\nabla }^2 \partial _t \hat{H}\Vert _{L^2((\tau ,T);L^2_2)} \lesssim \Vert h_0\Vert _{L^2_{p+1}}. \end{aligned} \end{aligned}$$

In order to derive estimates on derivatives of the next order, we invoke Lemma 5.9 with $k=1$,

5.27 $$\begin{aligned} \begin{aligned}&\Vert D_z\partial _t \hat{H}\Vert _{L^2(L^2_{2p})} + \Vert D_z\hat{\nabla }\hat{H}\Vert _{L^2(L^2)} + \Vert D_z\hat{\nabla }^2 \hat{H}\Vert _{L^2(L^2_2)} \\&\lesssim \Vert \hat{F}\Vert _{L^2(L^2_{2p})} + \Vert D_z \hat{F}\Vert _{L^2(L^2_{2p})} + \Vert \hat{G}\Vert _{L^2(L^2_{2p})} + \Vert D_z\hat{G}\Vert _{L^2(L^2_{2p})}. \end{aligned} \end{aligned}$$

Of course, our presentation here is a bit formal: Instead of considering derivatives $D_z$, we should more carefully apply difference quotients to the nonlinear equation. We have done so in Lemma 5.8 to deal with time derivatives. Because of the known reguarity in time (5.1), passing to the limit in the difference quotients don’t cause any problems, also in the nonlinear terms. The strategy remains the same for higher order derivatives in time, and we may generously simplify our presentation here by considering proper time derivatives in the sequel.

When it comes to higher order derivatives in tangential direction, a result analogous to (5.1) is missing, but will be derived by us in Corollary 5.12 below. We should thus be a bit more careful in our argumentation. For the sake of a simpler presentation though, we shall keep the notation $D_z$ and will tacitly interpret it as difference quotients in the tangential variables. Only in the discussion of the leading order nonlinear terms, we shall recall its actual meaning. For all other terms we will be rather formal.

Let us start considering the lower-order terms. From the definition of $\hat{G}$, we deduce that

$$\begin{aligned} \Vert \hat{G}\Vert _{L^2(L^2_{2p})} + \Vert D_z \hat{G}\Vert _{L^2(L^2_{2p})}&\lesssim \Vert \chi _{{{\,\textrm{spt}\,}}\psi } \hat{h}\Vert _{L^2(L^2)} + \Vert \chi _{{{\,\textrm{spt}\,}}\psi } \hat{\nabla }h\Vert _{L^2(L^2_2)}\\ {}&\quad + \Vert \chi _{{{\,\textrm{spt}\,}}\psi }D_z \hat{h}\Vert _{L^2(L^2)} + \Vert \chi _{{{\,\textrm{spt}\,}}\psi } D_z \hat{\nabla }\hat{h}\Vert _{L^2(L^2_2)}, \end{aligned}$$

where we have set $\psi = \zeta \hat{\eta }$.

We will now apply an interpolation to modify the weights on the right-hand side. Let $\phi$ be a cut-off function that is 1 in the support of $\psi$ and vanishes outside of a small neighborhood $\widehat{{{\,\textrm{spt}\,}}\psi }$ of this support. Then it holds for any regular function $\xi$ that

$$\begin{aligned} \int \phi ^2 \xi ^2 \, \hbox {d}\hat{x}&= \int {\left( \frac{\hbox {d}\hat{x}_n}{\hbox {d}\hat{x}_n}\right) } \phi ^2 \xi ^2\, \hbox {d}\hat{x}\\&= -2 \int \hat{x}_n \phi \xi \partial _n \xi \, \hbox {d}\hat{x} - 2\int \hat{x}_n \phi \partial _n \phi \xi ^2\, \hbox {d}\hat{x}. \end{aligned}$$

An application of the Cauchy–Schwarz inequalities then yields

$$\begin{aligned} \Vert \chi _{{{\,\textrm{spt}\,}}\psi }\xi \Vert _{L^2(L^2)} \lesssim \Vert \chi _{\widehat{{{\,\textrm{spt}\,}}\psi }} \xi \Vert _{L^2(L^2_2)} + \Vert \chi _{\widehat{{{\,\textrm{spt}\,}}\psi }} \hat{\nabla }\xi \Vert _{L^2(L^2_2)}. \end{aligned}$$

This argument can be repeated by writing $\hat{x}_n^q = \frac{1}{q+1} \frac{\hbox {d}}{\hbox {d}\hat{x}_n}\hat{x}_n^{q+1}$ and using that $\hat{\mu }_q\lesssim \hat{\mu }_2$ for $q\geqq 2$, to derive

5.28 $$\begin{aligned} \Vert \chi _{{{\,\textrm{spt}\,}}\psi }\xi \Vert _{L^2(L^2)} \lesssim \Vert \chi _{\widehat{{{\,\textrm{spt}\,}}\psi }} \xi \Vert _{L^2(L^2_q)} + \Vert \chi _{\widehat{{{\,\textrm{spt}\,}}\psi }} \hat{\nabla }\xi \Vert _{L^2(L^2_2)}, \end{aligned}$$

for any $q\in 2\textbf{N}$. By interpolation, this estimates extends to any $q>0$.

Making use of this interpolation-type estimate with suitable choices of *q* in the above estimate for $\hat{G}$ and estimating $\hat{\mu }_{2p}\lesssim \hat{\mu }_{p+1}\lesssim \hat{\mu }_{2} \lesssim 1$ yields

$$\begin{aligned} \Vert \hat{G}\Vert _{L^2(L^2_{2p})} + \Vert D_z \hat{G}\Vert _{L^2(L^2_{2p})}&\lesssim \Vert \chi _{\widehat{{{\,\textrm{spt}\,}}\psi }} \hat{h}\Vert _{L^2(L^2_{p+1})} + \Vert \chi _{\widehat{{{\,\textrm{spt}\,}}\psi }} \hat{\nabla }h\Vert _{L^2(L^2)}\\ {}&\quad + \Vert \chi _{\widehat{{{\,\textrm{spt}\,}}\psi }}\partial _t \hat{h}\Vert _{L^2(L^2_{2p})} + \Vert \chi _{\widehat{{{\,\textrm{spt}\,}}\psi }} \partial _t \hat{\nabla }\hat{h}\Vert _{L^2(L^2)}\\&\quad + \Vert \chi _{\widehat{{{\,\textrm{spt}\,}}\psi }} \hat{\nabla }^2 \hat{h}\Vert _{L^2(L^2_2)}, \end{aligned}$$

We may now invoke (5.26) with suitable choices of $\tau$, *T* and spatial cutoff *r* to deduce that

$$\begin{aligned} \Vert \hat{G}\Vert _{L^2(L^2_{2p})} + \Vert D_z \hat{G}\Vert _{L^2(L^2_{2p})} \lesssim \Vert h_0\Vert _{L^2_{p+1}}. \end{aligned}$$

It remains to estimate the nonlinear terms in (5.27). We promised to be more careful when considering higher order tangential difference quotients and we should thus briefly discuss the rigorous treatment of the leading order nonlinear terms. Because tangential derivatives leave the limit function $\hat{V}$ invariant in terms of scaling, cf. Lemma 5.3, these are terms of the order $\psi |d_i^s \hat{h}||\partial _t \hat{h}|$ and $\psi |\hat{h}||\partial _td_i^s \hat{h}|$, where $d_i^s$ is the difference quotient operator in direction $\hat{x}_i$, see also Lemma 5.8. By using the smallness of $\hat{H}$ in the assumption, the second of these terms can be absorbed into the left-hand side before passing to the limit $s\rightarrow 0$. The other term can be split into the two quadratic terms $\psi (d_i^s \hat{h})^2$ and $\psi (\partial _t \hat{h})^2$, among which we only have to consider the first one, because time regularity is already settled. Here, we notice that this term is bounded uniformly in *s*, because $\Vert \psi (\partial _i \hat{h})^2\Vert _{L^2_{2p}}$ can be estimated by $\Vert \hat{h}\Vert _{L^{\infty }} \Vert \psi \partial _i^2 \hat{h}\Vert _{L^2_{2}}$ plus lower order terms via the interpolation Lemma 5.7 and by using $\hat{\mu }_{2p}\lesssim \hat{\mu }_2$ in the support of $\hat{\eta }$. This term is controlled via (5.26). There are no further regularity issues popping up when considering higher order tangential derivatives. We shall continue with the rather formal discussion and summarize here that Lemma 5.10 implies

$$\begin{aligned}&\Vert \hat{F}\Vert _{L^2(L^2_{2p})} + \Vert D_z\hat{F}\Vert _{L^2(L^2_{2p})} \\&\quad \lesssim \varepsilon ^{\nu }\left( \Vert \psi \hat{h}\Vert _{L^2(L^2_{2p})} + {\Vert \psi D_z \hat{h}\Vert _{L^2(L^2_{2p})}} + {\Vert \psi D^2_z \hat{h}\Vert _{L^2(L^2_{2p})}} \right. \\&\qquad +\left. \Vert D_z \psi \hat{h}\Vert _{L^2(L^2_{2p})} + \Vert D_z \psi {D_z} \hat{h}\Vert _{L^2(L^2_{2p})}\right) \\&\quad \lesssim \Vert \chi _{{{\,\textrm{spt}\,}}\psi } \hat{h}\Vert _{L^2(L^2_{2p})} +\Vert \chi _{{{\,\textrm{spt}\,}}\psi } D_z \hat{h}\Vert _{L^2(L^2_{2p})} +\varepsilon ^{\nu } \Vert D^{2}_z \hat{H}\Vert _{L^2(L^2_{2p})}\\&\quad \lesssim \Vert h_0\Vert _{L^2_{p+1}} + \varepsilon ^{\nu } \Vert D_z \partial _t \hat{H}\Vert _{L^2(L^2_{2p})} + {\varepsilon ^\nu \Vert D_z \hat{\nabla }\hat{H}\Vert _{L^2(L^2)}}, \end{aligned}$$

where the last inequality is due to (5.26).

Plugging these estimates (for the lower-order terms involving $\hat{G}$ and the nonlinearities involving $\hat{F}$) into (5.27) thus yields

$$\begin{aligned} \Vert D_z\partial _t \hat{H}\Vert _{L^2(L^2_{2p})} + \Vert D_z\hat{\nabla }\hat{H}\Vert _{L^2(L^2)} + \Vert D_z\hat{\nabla }^2 \hat{H}\Vert _{L^2(L^2_2)} \lesssim \Vert h_0\Vert _{L^2_{p+1}}, \end{aligned}$$

provided that $\varepsilon ^\nu$ is chosen small enough that the final terms above which it multiplies can be absorbed into the left hand side.

This procedure can be iterated, with a suitable adaption of $\tau , T$ and the radii *r* of the spatial cut-off functions $\eta$ in each step to prove that

$$\begin{aligned} \Vert D_z^k\partial _t \hat{H}\Vert _{L^2(L^2_{2p})} + \Vert D_z^k\hat{\nabla }\hat{H}\Vert _{L^2(L^2)} + \Vert D_z^k\hat{\nabla }^2 \hat{H}\Vert _{L^2(L^2_2)} \lesssim \Vert h_0\Vert _{L^2_{p+1}}, \end{aligned}$$

inductively. This implies the desired bounds.

Finally, we use generalized Sobolev embeddings to pass from $L^2$ to $L^\infty$ estimates.

### Corollary 5.12

(Tangential nonlinear smoothing in weighted uniform norms) Let $\hat{H}$ be the solution to the transformed equation (5.23) and suppose that $\Vert \hat{H}\Vert _{L^{\infty }}\leqq \varepsilon$ for some $\varepsilon$ small enough. Let $0<\tau<1<T$ be given. Then, for any $k\in \textbf{N}_0$, it holds that

$$\begin{aligned} \Vert D_z^k \hat{H}\Vert _{L^{\infty }(L^{\infty })} + \Vert \hat{x}_n D_z^k \hat{\nabla }\hat{H}\Vert _{L^{\infty }(L^{\infty })}\lesssim \Vert h_0\Vert _{L^2_{p+1}}. \end{aligned}$$

### Proof

The estimate basically follows from Proposition 5.11 via generalized Sobolev embeddings using compact support in the $z=(t,x')\in {\textbf{R}}^n$ variables, followed by (two) integrations in $x_n$ where we have a vanishing boundary condition at one end $x_n=r$ only. Indeed, for $m\in \textbf{N}$ with $m>{n/2}$, it holds that

$$\begin{aligned} \Vert D_z^k\hat{H}\Vert _{L^{\infty }(L^{\infty })}&\lesssim \sum _{\ell =0}^m \Vert D_z^{k+\ell }\hat{H}\Vert _{L^{2}(L^{2})} +\sum _{\ell =0}^m \Vert D_z^{k+\ell }\partial _{\hat{x}_n}\hat{H}\Vert _{L^{2}(L^{2})}. \end{aligned}$$

We now use the Hardy inequality

$$\begin{aligned} \Vert \xi \Vert _{L^2(L^2)} \lesssim \Vert \partial _{\hat{x}_n}\xi \Vert _{L^2(L^2_2)}, \end{aligned}$$

whose proof is similar to that of (5.28), to eliminate the zero-order terms on the right-hand side, thus,

$$\begin{aligned} \Vert D_z^k\hat{H}\Vert _{L^{\infty }(L^{\infty })} \lesssim \sum _{\ell =0}^{m+1} \Vert D_z^{k+\ell }\hat{\nabla }\hat{H}\Vert _{L^{2}(L^{2})}. \end{aligned}$$

Finally, we apply Proposition 5.11 to infer the desired control of the first term in the statement of the lemma. The second term is bounded analogously, by applying the same argument to $\hat{x}_n D^k \hat{\nabla }\hat{H}$ in place of $D^k \hat{H}$.

We are now well-prepared to prove Proposition 4.4.

### Proofs of Proposition 4.4

As a consequence of Corollary 5.12 and the construction of $\hat{H}$, we find for any $x_0\in \partial \Omega$, $0<\tau<1<T$ and any $r>0$ small enough that

$$\begin{aligned} \Vert \partial _t^k h\Vert _{L^{\infty }((\tau ,T)\times B_r(x_0))} \lesssim \Vert h_0\Vert _{L^2_{p+1}}. \end{aligned}$$

In particular, covering a small band along the domain boundary with a finite number of balls, the latter extends to the band $\Omega _r = \{x\in \Omega :{{\,\textrm{dist}\,}}(x,\partial \Omega )\leqq r\}$ for some small $r>0$,

$$\begin{aligned} \Vert \partial _t^k h\Vert _{L^{\infty }((\tau ,T)\times \Omega _r)} \lesssim \Vert h_0\Vert _{L^2_{p+1}}. \end{aligned}$$

As mentioned earlier, similar (but simpler, thanks to the strict parabolicity) arguments in the interior of the domain $\Omega$ yield analogous estimates on $\Omega \setminus \bar{\Omega }_r$. Both together prove the statement of the proposition.

## Proof of Second Dichotomy

In this final section, we turn to the proof of Theorem 3.4, which states optimal exponential convergence of the relative error under the assumption that *V* is an ordinary limit in the sense of Definition 3.3. Thanks to our first dichotomy result—Theorem 3.1—and Proposition 4.4, it is enough to establish convergence at some exponential rate, which is the main result of the present section.

### Theorem 6.1

(Ordinary limits are approached exponentially fast) Under the hypotheses of Theorem 3.1, if *V* is an ordinary limit of the dynamics (1.2), the convergence takes place exponentially fast, i.e., there exists a rate $\gamma >0$ such that

$$\begin{aligned} \begin{aligned} \Vert h(t)\Vert _{L^2_{p+1}} =O(e^{-\gamma t}) {\quad \text{ as } \quad t \rightarrow \infty .} \end{aligned}\end{aligned}$$

The proof of exponential convergence relies on Choi and Sun’s refinement—Lemma 4.3—of the Merle–Zaag dynamical systems result recalled above. It roughly says that if a solution is known to be small on a large time interval, then, up to a possible error caused by the neutral modes, the stable and unstable modes should be much smaller than an exponentially decaying term in the middle of this time interval. (Note the unstable modes tend to decay like the stable ones if time goes backward and this is why we need to go to the middle of time interval.) The main issue to take care of is thus the control of the neutral modes.

The underlying idea for controlling the neutral modes is reducing the amplitude of the neutral modes by changing the reference stationary solution in the direction of the neutral modes. This can be effectively done if the limit *V* is ordinary. This strategy goes back to the work of Allard and Almgren [3], who gave kernel integrability conditions guaranteeing that minimal surfaces converge to their tangent cones sequentially and exponenentially fast. See also Section 6 of Simon [49], or the recent contributions of Choi, Choi, Kim and Sun in various combinations [17, 20].

In order to pursue this strategy, we have to prove that being an ordinary limit is an open property among stationary solutions S. This crucial insight requires some technical preparations.

### Lemma 6.2

(Lower semicontinuity of kernel dimension at an ordinary limit) Let $V\in S$ be an ordinary limit and $\delta >0$ as in Definition 3.3. Let $\tilde{V} \in S$ be sufficiently close that $h = {V}/{\tilde{V}}-1$ satisfies $\Vert h\Vert _{L^\infty } \leqq \delta$, so that $h=\Phi _V(\psi )$ for some $\psi \in \ker L_V$. Then it holds that

6.1 $$\begin{aligned} \frac{V}{\tilde{V}}(\hbox {d}\Phi _V)_\psi (\ker L_V ) \subset \ker L_{\tilde{V}}. \end{aligned}$$

### Proof

Let $0 \in \mathcal {U}\subset \ker L_V$ the neighbourhood provided by Definition 3.3. We fix another element in the kernel, $\zeta \in \ker L_V$, and choose $s_0$ small enough such that $\psi + s\zeta \in \mathcal {U}$ for any $s\in (-s_0,s_0)$. Then $h_s = \Phi _V(\psi +s\zeta )$ defines a family of stationary relative errors (3.7) with $h_0 = h$, or equivalently, $\tilde{V}_s = V(h_s+1) \in S$ defines a stationary solution in terms of the original variables with $\tilde{V}_0=\tilde{V}$. Changing the reference stationary solution $\tilde{h}_s = \tilde{V}_s/\tilde{V}-1$ solves $\tilde{h}_0=0$ and

$$\begin{aligned} L_{\tilde{V}}\tilde{h}_s = M(\tilde{h}_s). \end{aligned}$$

We may now rewrite

$$\begin{aligned} \tilde{h}_s = \frac{V}{\tilde{V}}\left( \Phi _V(\psi +s\zeta ) - \Phi _V(\psi )\right) , \end{aligned}$$

and thanks to the regularity properties of the diffeomorphism $\Phi _V$, differentiation in the previous two identities yields

$$\begin{aligned} L_{\tilde{V}} \left( \frac{V}{\tilde{V}}( \hbox {d}\Phi _V)_{\psi }\zeta \right) = L_{\tilde{V}} \left. \partial _s \right| _{s=0} \tilde{h}_s = M'(0) \left. \partial _s \right| _{s=0} \tilde{h}_s=0, \end{aligned}$$

since $M(h)=O(h^2)$ as $h\rightarrow 0$ in (3.7). The latter verifies the inclusion (6.1).

The next lemma guarantees that spectral gaps are preserved by nearby stationary solutions.

### Lemma 6.3

(Continuity of spectral gap and nullity) Let $V \in S$ be an ordinary limit and let the sequence $\{V_{\ell }\}_{\ell \in \textbf{N}} \in S$ of stationary solutions converge to *V* relatively-uniformly, meaning $h_{\ell } = V_{\ell }/V-1$ satisfies

$$\begin{aligned} \Vert h_{\ell }\Vert _{L^{\infty }}\rightarrow 0\quad \text{ as } \ell \rightarrow \infty . \end{aligned}$$

Let $\{\phi _{\ell }\}_{\ell \in \textbf{N}}$ denote a sequence of normalized eigenfunctions, i.e.,

$$\begin{aligned} L_{V_{\ell }}\phi _{\ell } = \lambda _{\ell }\phi _{\ell },\quad \text{ and }\quad \int \phi _{\ell }^2 V_{\ell }^{p+1}\, \textrm{d}x = 1, \end{aligned}$$

for some $\lambda _{\ell }\in {\textbf{R}}$. Suppose that the sequence of eigenvalues is bounded, $|\lambda _{\ell }|\leqq \Lambda$ for some $\Lambda >0$. Then there exists a subsequence $\{\phi _{\ell _k}\}_{k\in \textbf{N}}$ and a function $\phi \in L^2(V^{p+1}\textrm{d}x)$ such that

$$\begin{aligned} \Vert \phi _{\ell _k}-\phi \Vert _{L^2(V^{p+1}\textrm{d}x)} \rightarrow 0\quad \text{ as } k\rightarrow \infty . \end{aligned}$$

The limiting function $\phi$ is a normalized eigenfunction, i.e,

$$\begin{aligned} L_{V}\phi = \lambda \phi ,\quad \text{ and }\quad \int \phi ^2 V^{p+1}\, \textrm{d}x = 1, \end{aligned}$$

for some $\lambda \in {\textbf{R}}$. Moreover, the following hold true:

1. If $\lambda _{\ell }=0$ for all $\ell \in \textbf{N}$, then $\lambda =0$.
2. If $\lambda _{\ell }>0$ for all $\ell \in \textbf{N}$, then $\lambda >0$.
3. If $\lambda _{\ell }<0$ for all $\ell \in \textbf{N}$, then $\lambda <0$.

The lemma entails, in particular, that if $|\lambda _{\ell }|>0$ for all $\ell \in \textbf{N}$, then

$$\begin{aligned} \liminf _{\ell \rightarrow \infty } |\lambda _{\ell }| \geqq \min \{ -\lambda _u, \lambda _s\}, \end{aligned}$$

where $\lambda _u$ is the largest negative and $\lambda _s$ is the smallest positive eigenvalue of $L_V$.

### Proof

For the compactness assertion, we aim to bound $\sup _\ell \Vert \phi _\ell \Vert _{H^1}$. Applying Proposition 5.5 with $h/t =\phi _\ell =f/(\lambda _\ell t + 1)$ yields the unweighted gradient bound

$$\begin{aligned} \Vert \nabla \phi _{\ell } \Vert _{L^2} \lesssim (\lambda _\ell +1) \Vert \phi _{\ell }\Vert _{L^2_{2p}} + \Vert \phi _{\ell }\Vert _{L^2_{p+1}} \lesssim (\lambda _\ell +2) \Vert \phi _{\ell }\Vert _{L^2_{p+1}} \leqq \Lambda +2. \end{aligned}$$

Now the local independence (in the relatively-uniform topology) of constants in $\lesssim$ on $V_\ell \in S$ combines with Lemma 5.2 to imply the sequence $\phi _\ell$ is bounded in the Sobolev space $H^1(\Omega )$. Via a Rellich compactness argument, we conclude that $\{\phi _{\ell }\}_{\ell \in \textbf{N}}$ converges strongly subsequentially in $L^2$ (and then also in $L^2(V^{p+1}\hbox {d}x)$) and weakly in $H^1(V^2\hbox {d}x)$ towards some function $\phi$. Moreover, thanks to the Bolzano–Weierstraß theorem, we find that the sequence of eigenvalues $\{\lambda _{\ell }\}_{\ell \in \textbf{N}}$ converges subsequentially to some $\lambda \in {\textbf{R}}$.

Considering a common subsequence (indexed by $\ell _{k}$) and passing to the limit in the weak formulation of the eigenvalue equation,

$$\begin{aligned} \int \nabla \phi _{\ell _k}\cdot \nabla f V_{\ell _k}^2\, \hbox {d}x =\left( \lambda _{\ell _k} + p-1\right) \int \phi _{\ell _k} f V_{\ell _k}^{p+1}\, \hbox {d}x, \end{aligned}$$

where we also use the uniform convergence of the relative error $h_{k}$, we find that $\phi$ is a normalized eigenfunction of $L_V$ with eigenvalue $\lambda$.

It remains to derive the assertion on the sign of the limiting eigenvalues. The first statement is trivial, while the proofs of two others are identical. Let’s thus focus on one of them, say the middle one. It is clear that the limiting eigenvalue is nonnegative, $\lambda \geqq 0$ and we have to rule out that it is in fact zero. For this purpose, we note that the eigenfunctions $\phi _{\ell }$ are orthogonal to the kernel,

6.2 $$\begin{aligned} \int \phi _{\ell } \zeta _{\ell } V_{\ell }^{p+1}\, \hbox {d}x = 0\quad \text{ for } \text{ any } \zeta _{\ell }\in \ker L_{V_\ell }. \end{aligned}$$

We pick $\zeta \in \ker L_V$ and define $\zeta _{\ell }\in \ker L_{V_{\ell }}$ according to the inclusion (6.1) derived in Lemma 6.2 by

$$\begin{aligned} \zeta _{\ell } = \frac{1}{h_{\ell }+1}(\hbox {d}\Phi _V)_{\psi _{\ell }} \zeta , \end{aligned}$$

where $\psi _{\ell }\in \ker L_V$ is such that $h_{\ell } = \Phi _V(\psi _{\ell })$. It is straightforward to verify that $\zeta _{\ell }$ converges to $\zeta$ strongly in $L^2(V^{p+1}\hbox {d}x)$ as $\ell \rightarrow \infty$. Indeed, because of the imposed convergence of the relative error $h_{\ell }$ and since the diffeomorphism $\Phi _V$ vanishes only at the origin, we must have that $\psi _{\ell }\rightarrow 0$ strongly in $L^2(V^{p+1})$. Furthermore, by the continuity of the derivative $\hbox {d}\Phi _V$, it holds that $(\hbox {d}\Phi _V)_{\psi _{\ell }}\rightarrow \textrm{id}$. Using once again the uniform convergenve of the relative error, we conclude that $\zeta _{\ell }\rightarrow \zeta$ in $L^2(V^{p+1}\hbox {d}x)$.

We now pass to the limit in the orthogonality condition (6.2) with our particular construction of the $\zeta _{\ell }$’s, which was arbitrary in the choice of $\zeta$, and find

$$\begin{aligned} \int \phi \zeta V^{p+1}\, \hbox {d}x =0\quad \text{ for } \text{ any } \zeta \in \ker L_V. \end{aligned}$$

Hence, $\phi$ is a (nontrivial) eigenfunction of $L_V$ that is orthogonal to the kernel. We conclude that $\lambda >0$ as desired.

The preceeding analysis allows us to conlude quite easily that the dimension of the kernels of the linear operators remains constant if the reference stationary solution is changed in a neighborhood of an ordinary limit *V*.

### Lemma 6.4

(Invariance of the kernel dimension near ordinary limits) Let $V\in S$ be an ordinary limit and $\delta >0$ as in Definition 3.3. Let $\tilde{V}\in S$ be a stationary solution close to *V* in the sense that $h = \tilde{V}/V-1$ satisfies $\Vert h\Vert _{L^{\infty }}\leqq \tilde{\delta }$, for some $\tilde{\delta }\in (0,\delta )$. Then $\tilde{\delta }$ sufficiently small implies

$$\begin{aligned} \dim \ker L_V = \dim \ker L_{\tilde{V}}. \end{aligned}$$

### Proof

As a consequence of Lemma 6.2, and because $(\hbox {d}\Phi _V)_{\psi }$ is an isomorphism, it is clear that

6.3 $$\begin{aligned} K= \dim \ker L_V \leqq \dim \ker L_{\tilde{V}}. \end{aligned}$$

We argue that both kernels have indeed the same dimension if $\tilde{\delta }$ is sufficiently small. We give an indirect argument and derive a contradiction by assuming that there exists a sequence $h_{\ell } = V_{\ell }/V-1$ satisfying $\Vert h_{\ell }\Vert _{L^{\infty }}\leqq \frac{1}{\ell }$ and $\dim \ker L_{ V_{\ell }}\geqq K+1$. We pick $K+1$ orthonormal (and thus linearly independent) functions $\phi _{\ell ,1},\dots ,\phi _{\ell ,K+1}$ in $\ker L_{ V_{\ell }}$.

By the virtue of Lemma 6.3, there exist subsequences (that we will not relabel) and normalized functions $\phi _1,\dots ,\phi _{K+1}$ in $\ker L_V$ such that

$$\begin{aligned} \phi _{\ell ,k}\rightarrow \phi _k\quad \text{ in } L^2(V^{p+1}) \text{ as } \ell \rightarrow \infty . \end{aligned}$$

In particular, using in addition that $\{h_{\ell }\}_{\ell \in \textbf{N}}$ is converging uniformly, we find that $\phi _1,\dots ,\phi _{K+1}$ is orthonormal. Thus, the dimension of $\ker L_V$ is at least $K+1$, which contradicts (6.3).

Collecting these technical preparations, we are able to derive the aforementioned key feature: that ordinariness is a relatively-uniformly open property in the set *S* of stationary limits.

### Proposition 6.5

(Ordinary limits form an open subset of *S*) Let $V \in S$ be an ordinary limit. If $\tilde{V} \in S$ is a stationary solution sufficiently close to *V* in the relatively-uniform topology, then $\tilde{V}$ is also an ordinary limit.

### Proof

By combining the results from Lemmas 6.2 and 6.4, we see that

$$\begin{aligned} \frac{\tilde{V}}{V} \ker L_{\tilde{V}} = (d \Phi _V)_\psi (\ker L_V), \end{aligned}$$

where $\psi \in \ker L_V$ is such that $h = \Phi _V(\psi )$. It follows that the mapping $\Phi _{\tilde{V}}$ defined by

$$\begin{aligned} \Phi _{\tilde{V}}(\tilde{\psi }):=\frac{V}{\tilde{V}} \left[ \Phi _V\left( \psi + ((\hbox {d}\Phi _V)_\psi )^{-1} (\tfrac{\tilde{V}}{V} \tilde{\psi }) \right) -\Phi _V(\psi ) \right] ,\end{aligned}$$

for $\tilde{\psi }\in \ker {L_{\tilde{V}}}$, is a $C^1$ diffeomorphism from a neighborhood of $0\in \ker L_{\tilde{V}}$ into the set of stationary solutions $L_{\tilde{V}}\tilde{h} = M(\tilde{h})$ and satisfies all the properties listed in Definition 3.3, as can be readily verfied. Notice also that we have see the construction of this diffeomorphism already in the proof of Lemma 6.2: If $\zeta \in \ker L_V$ is sufficiently small so that $h_{\zeta } = \Phi _V(\psi + \zeta )$ is well-defined, then $V_{\zeta } = V(h_{\zeta }+1)$ is a stationary solution and the relative error $\tilde{h}_{\zeta }$ with respect to $\tilde{V}$,

$$\begin{aligned} \tilde{h}_{\zeta } = \frac{V_{\zeta }}{\tilde{V}} - 1 = \frac{V}{\tilde{V}}\left( h_{\zeta } - \frac{\tilde{V}}{V}+1\right) = \frac{V}{\tilde{V}}\left( \Phi _V(\psi +\zeta ) - \Phi _V(\psi )\right) , \end{aligned}$$

solves the stationary error equation relative to the reference point $\tilde{V}$.

We will now derive a building block for the proof of Theorem 6.1 which exploits both the previous Proposition 6.5 and the Choi-Sun refinement of the Merle–Zaag Lemma 4.3, to yield a suitable reduction of the neutral modes as announced at the beginning of this section. The actual proof of exponential convergence will follow subsequently by iteration.

### Proposition 6.6

(Improvement by changing reference stationary solutions) Let $V\in S$ be an ordinary limit (1.3). There exist four constants $\varepsilon _0,\delta _0\in (0,1)$ and $\tau ,C\in (1,\infty )$ with the following property: Let $V(1+h(t))$ solve the dynamics (1.2) and stay near *V*,

6.4 $$\begin{aligned} \sup _{t\geqq 0} \Vert h(t)\Vert _{L^{\infty }}\leqq \delta _0; \end{aligned}$$

$\{V(1+g_s)\}_{s\geqq 0} {\subset S}$ be a family of stationary solutions to (1.3) also close to *V*,

6.5 $$\begin{aligned} \sup _{s\geqq 0} \Vert g_s \Vert _{L^{\infty }} \leqq \delta _0; \end{aligned}$$

and suppose $V(1+h(t))$ is $\varepsilon$-close to $V(1+g_s)$ on $t\in [s,s+2]$,

6.6 $$\begin{aligned} \sup _{t\in [s,s+2]}\Vert h(t) -g_s \Vert _{L^2_{p+1}} \leqq \varepsilon \end{aligned}$$

for all $s\geqq 0$ for some $\varepsilon \leqq \varepsilon _0$.

Then there is another family of stationary solutions $\{V(1+\hat{g}_s)\}_{s\geqq \tau }$ so that $V(1+h(t))$ is $\tfrac{\varepsilon }{2}$-close to $V(1+\hat{g}_s)$ on $t\in [s,s+2]$

6.7 $$\begin{aligned} \sup _{t\in [s,s+2]}\Vert h(t) -\hat{g}_s \Vert _{L^2_{p+1}} \leqq \frac{\varepsilon }{2} \end{aligned}$$

for all $s\geqq \tau$. Moreover, for each $s\geqq 0$, $\hat{g}_{s+\tau }$ is close enough to $g_s$ that

6.8 $$\begin{aligned} \Vert g_s -\hat{g}_{s+\tau } \Vert _{L^\infty } \leqq C \varepsilon . \end{aligned}$$

In the proof of Theorem 6.1 below, the exponential convergence rate $\gamma ={\frac{\log 2}{\tau }}$ is determined by the delay $\tau$ provided by the preceding prop.

### Proof

To simplify the notation, we write

$$\begin{aligned}\Vert f\Vert _{\tilde{V}} = \Vert f \Vert _{L^2(\tilde{V}^{p+1}) }\end{aligned}$$

for any $\tilde{V}$ stationary solution (1.3). We start by noting that for two stationary solutions $\tilde{V} = V(1+\tilde{g})$ and $\hat{V} = V(1+\hat{g})$ to (1.3) with $\Vert \tilde{g} \Vert _{L^\infty }$, $\Vert \hat{g} \Vert _{L^\infty }\leqq \delta _0<1$, there holds

$$\begin{aligned} \Vert f \Vert _{\tilde{V}} \leqq \left( \frac{1+ \delta _0}{1-\delta _0}\right) ^{\frac{p+1}{2}} \Vert f\Vert _{\hat{V}}.\end{aligned}$$

i.e., two norms are equivalent

$$\begin{aligned}\Vert f \Vert _{\tilde{V} } \lesssim \Vert f \Vert _{\hat{V}} \end{aligned}$$

provided $\delta _0<1/2$. Throughout this proof, $f \lesssim g$ denotes the inequality $f\leqq C g$ for some constant *C* which may depend on *n*, *m*, and *V* but uniform in small $\varepsilon _0$, $\delta _0$, and large $\tau$.

It suffices to show how $\hat{g} _\tau$ is chosen so to satisfy (6.7) and (6.8). For other $\hat{g}_{s'+\tau }$, $s'>0$, we may shift the time of original problem by $s'$, consider $V(1+h(s'+t))$ and $V(1+g_{s'+s})$ in place of $V(1+h(t))$ and $V(1+g_s)$, respectively, and re-apply the previous assertion. We now notice that by the triangle inequality and the hypothesis in (6.6), we have that

$$\begin{aligned} \Vert g_{s+2}-g_{s}\Vert _V \leqq \Vert g_{s+2}-h_{s+1}\Vert _V + \Vert h_{s+1}-g_{s}\Vert _V \leqq 2\varepsilon , \end{aligned}$$

for any $s\geqq 0$, and thus by iteration,

6.9 $$\begin{aligned} \Vert g_{2k} - g_0\Vert _V \leqq k\varepsilon , \end{aligned}$$

for any $k\in \textbf{N}_0$. By another application of the triangle inequality on (6.6) and (6.9), it holds that, for $\tau >1$,

6.10 $$\begin{aligned} \sup _{t \in [0,2\tau ]}\Vert h(t)-g_0\Vert _V \lesssim \tau \varepsilon .\end{aligned}$$

Let us denote $\tilde{V}:= V(1+g_0)$, a new stationary solution. If we write the solution $V(1+h(t))$ to (1.2) in terms of $\tilde{V}$ and its relative quantity by $V(1+h(t))= \tilde{V}(1+\tilde{h}(t))$, then $\tilde{h}(t):= \frac{h(t)-g_0}{1+g_0}$. Note that $\tilde{h}(t)$ solves the evolution equation for relative error

6.11 $$\begin{aligned} \partial _t \tilde{h} + L_{\tilde{V}}\tilde{h} = N_{\tilde{V}}(\tilde{h}), \end{aligned}$$

equation (2.1) with new refenence stationary solution $\tilde{V}$.

In view of (6.4), (6.5), (6.10), and the equivalence between $\Vert \cdot \Vert _{V}$ and $\Vert \cdot \Vert _{\tilde{V}}$, observe that

6.12 $$\begin{aligned} \sup _{t\geqq 0}\Vert \tilde{h}(t) \Vert _{L^\infty } \leqq \frac{2\delta _0 }{1-\delta _0}\quad \text { and } \quad \sup _{t\in [0,2\tau ] }\Vert \tilde{h}(t)\Vert _{\tilde{V}} \lesssim \tau \varepsilon . \end{aligned}$$

If we choose $\delta _0$ sufficiently small, then the smoothing estimate in Proposition 4.4 applies to $\tilde{h}(t)$, a solution to (6.11), and we have

6.13 $$\begin{aligned} \sup _{t\in [1,2\tau ] }\Vert \tilde{h}(t)\Vert _{L^\infty }+ \Vert \partial _t \tilde{h}(t) \Vert _{L^\infty } \lesssim \varepsilon \tau .\end{aligned}$$

(In fact, the constants $\varepsilon$ and *C* in Proposition 4.4 depend on $\tilde{V}$, not *V*. Since $\tilde{V}= V(1+g_0)$ and $\Vert g_0 \Vert _{L^\infty }\leqq \delta _0$, by assuming $\delta _0$ is small, we may assume $\varepsilon$ and *C* do not depend on the choice of $g_0$.)

In the next step, we aim at applying a Merle–Zaag-type lemma to the unstable, center, and stable modes of $\tilde{h}$. For this purpose, we introduce the $L^2(\tilde{V}^{p+1}\hbox {d}x)$ orthogonal projections $\tilde{P}_u$, $\tilde{P}_u$ and $\tilde{P}_s$ onto the unstable, center, and stable eigenspaces generated by $L_{\tilde{V}}$, and write $\tilde{h}_u = \tilde{P}_u\tilde{h}$, $\tilde{h}_c = \tilde{P}_c\tilde{h}$ and $\tilde{h}_s = \tilde{P}_s\tilde{h}$. Arguing similarly to the proof of Theorem 3.1, we derive the system of ordinary differential equations

$$\begin{aligned} \frac{\hbox {d}}{\hbox {d}t} \Vert \tilde{h}_u\Vert _{\tilde{V}}+ \tilde{\lambda }_u \Vert \tilde{h}_u\Vert _{\tilde{V}}&\geqq -C\varepsilon \tau \left( \Vert \tilde{h}_u\Vert _{\tilde{V}}+ \Vert \tilde{h}_c\Vert _{\tilde{V}} + \Vert \tilde{h}_s\Vert _{\tilde{V}}\right) ,\\ \left| \frac{\hbox {d}}{\hbox {d}t} \Vert \tilde{h}_c\Vert _{\tilde{V}} \right|&\leqq C\varepsilon \tau \left( \Vert \tilde{h}_u\Vert _{\tilde{V}}+ \Vert \tilde{h}_c\Vert _{\tilde{V}} + \Vert \tilde{h}_s\Vert _{\tilde{V}}\right) ,\\ \frac{\hbox {d}}{\hbox {d}t} \Vert \tilde{h}_u\Vert _{\tilde{V}}+ \tilde{\lambda }_s \Vert \tilde{h}_u\Vert _{\tilde{V}}&\leqq C\varepsilon \tau \left( \Vert \tilde{h}_u\Vert _{\tilde{V}}+ \Vert \tilde{h}_c\Vert _{\tilde{V}} + \Vert \tilde{h}_s\Vert _{\tilde{V}}\right) , \end{aligned}$$

for all $t\in [1,2\tau ]$, for some constant $C>0$, where $\tilde{\lambda }_u$ is the largest negative and $\tilde{\lambda }_s$ the smallest positive eigenvalue of $L_{\tilde{V}}$. Note that $\varepsilon _0>0$ and $\tau >0$ are not fixed yet. Let us denote $\sigma :=C\varepsilon \tau$. Suppose we choose $\varepsilon _0$ and $\tau$ so that

$$\begin{aligned} \sigma \leqq C \varepsilon _0 \tau \leqq \tilde{\lambda }:= \frac{1}{2} \min \{-\tilde{\lambda }_u,\tilde{\lambda }_s\},\end{aligned}$$

then the system implies

$$\begin{aligned} \frac{\hbox {d}}{\hbox {d}t} \Vert \tilde{h}_u\Vert _{\tilde{V}}- \tilde{\lambda }\Vert \tilde{h}_u\Vert _{\tilde{V}}&\geqq -\sigma \left( \Vert \tilde{h}_c\Vert _{\tilde{V}} + \Vert \tilde{h}_s\Vert _{\tilde{V}}\right) ,\\ \left| \frac{\hbox {d}}{\hbox {d}t} \Vert \tilde{h}_c\Vert _{\tilde{V}} \right|&\leqq \sigma \left( \Vert \tilde{h}_u\Vert _{\tilde{V}}+ \Vert \tilde{h}_c\Vert _{\tilde{V}} + \Vert \tilde{h}_s\Vert _{\tilde{V}}\right) ,\\ \frac{\hbox {d}}{\hbox {d}t} \Vert \tilde{h}_u\Vert _{\tilde{V}}+ \tilde{\lambda }\Vert \tilde{h}_u\Vert _{\tilde{V}}&\leqq \sigma \left( \Vert \tilde{h}_u\Vert _{\tilde{V}}+ \Vert \tilde{h}_c\Vert _{\tilde{V}} \right) , \end{aligned}$$

for any $t\in [1,2\tau ]$. By the virtue of Lemma 4.3 and the bound in (6.12), there exits a constant $\sigma _0$ dependent only on $\tilde{\lambda }$ such that if we further assume $C\varepsilon _0 \tau \leqq \sigma _0$, it holds that

6.14 $$\begin{aligned} \Vert \tilde{h}_u(t)\Vert _{\tilde{V}} + \Vert \tilde{h}_s(t)\Vert _{\tilde{V}} \lesssim \varepsilon \tau \Vert \tilde{h}_c(t)\Vert _{\tilde{V}} + \varepsilon \tau e^{-\frac{1}{8}\tilde{\lambda }\tau }\lesssim (\varepsilon \tau )^2 + \varepsilon \tau e^{-\frac{1}{8}\tilde{\lambda }\tau }, \end{aligned}$$

for any $t\in [\frac{3\tau }{4},\frac{5\tau }{4}]$. Here, we applied the lemma on the interval $t\in [ \frac{\tau }{2},\frac{3\tau }{2} ]$. We remark that as a direct consequence of Lemma 6.3, $\tilde{\lambda }$ can be bounded away from zero uniformly in $\tilde{V}$, more precisely, we can suppose that

6.15 $$\begin{aligned} \tilde{\lambda }> \frac{1}{2} \min \{-\lambda _u, \lambda _s\} \end{aligned}$$

if $\delta _0$ is chosen sufficiently small.

We still have to bound the center modes. For this, we make use of the fact that, by the virtue of Proposition 6.5 and our hypothesis in (6.5), the limit $\tilde{V} = V(g_s+1)$ is ordinary. We denote by $\Phi _{\tilde{V}}$ the diffeomorphism between suitable subsets of $\ker L_{\tilde{V}}$ and the set of stationary solutions relative to $\tilde{V}$ described in Definition 3.3. Thanks to the bound in (6.12) that $\Vert \tilde{h}_c(\tau )\Vert _{\tilde{V}} \leqq \Vert \tilde{h}(\tau )\Vert _{\tilde{V}} \lesssim \varepsilon \tau$, for any small $\hat{\delta }$, if $\varepsilon _0 \tau$ is chosen sufficiently small, there exists a function $\tilde{g}_\tau = \Phi _{\tilde{V}}(\tilde{h}_c(\tau ))$ with $\Vert \tilde{g}_\tau \Vert _{L^{\infty }}\leqq \hat{\delta }$ solving $L_{\tilde{V}}(\tilde{g}_\tau ) = M(\tilde{g}_\tau )$ (i.e., $\tilde{V}(1+\tilde{g}_\tau )$ solves (1.3).) Moreover, since $\Phi _{\tilde{V}}(0)=0$ and $(\hbox {d}\Phi _{\tilde{V}})_0=\textrm{id}$, we have that

6.16 $$\begin{aligned} \begin{aligned} \Vert \tilde{g}_\tau - \tilde{h}_c(\tau )\Vert _{\tilde{V}}&= \Vert \Phi _{\tilde{V}}(\tilde{h}_c(\tau )) - \Phi _{\tilde{V}}(0) - (\hbox {d}\Phi _{\tilde{V}})_0(\tilde{h}_c(\tau ))\Vert _{\tilde{V}} \\ {}&= o(\Vert \tilde{h}_c(\tau )\Vert _{\tilde{V}}) = o(\varepsilon \tau ). \end{aligned}\end{aligned}$$

In order to observe that this estimate is stable under-order-one variations in time, we recall that the center modes solve the (finite dimensional) system $\partial _t \tilde{h}_c = \tilde{P}_c N_{\tilde{V}}(\tilde{h})$. An integration over some interval $[\tau ,t]$ and the quadratic estimate (4.3) on the nonlinearity give

$$\begin{aligned} \Vert \tilde{h}_c(t) -\tilde{h}_c(\tau )\Vert _{\tilde{V}} \leqq \int _{\tau }^t \Vert N_{\tilde{V}}(\tilde{h})\Vert _{\tilde{V}}\, \hbox {d}t \lesssim \int _{\tau }^t\left( \Vert \tilde{h}\Vert _{L^{\infty }} + \Vert \partial _t \tilde{h}\Vert _{L^{\infty }}\right) \Vert \tilde{h}\Vert _{\tilde{V}}\, \hbox {d}t. \end{aligned}$$

We apply the bound in (6.12) and (6.13) to conclude that

$$\begin{aligned} \sup _{ t\in [\tau ,\tau +2 ]} \Vert \tilde{h}_c(t)-\tilde{h}_c(\tau )\Vert _{\tilde{V}} \lesssim (\varepsilon \tau )^2,\end{aligned}$$

and thus, (6.16) can be generalized to

6.17 $$\begin{aligned} \sup _{t\in [\tau ,\tau +2]}\Vert \tilde{g}_{\tau } - \tilde{h}_c(t)\Vert _{\tilde{V}} = o(\varepsilon \tau ). \end{aligned}$$

Later when we show (6.8), it will be necessary to quantify the relation between $\hat{\delta }$ and $\varepsilon \tau$. For this purpose, we notice that since $\tilde{g}_\tau$ solves $L_{\tilde{V}}\tilde{g}_\tau = M(\tilde{g}_\tau )$, it satisfies the estimate $\Vert \tilde{g}_\tau \Vert _{L^{\infty }} \lesssim \Vert \tilde{g}_\tau \Vert _{\tilde{V}}$, and the inequality is uniform in $\tilde{V}$ and depends only on the bound in (6.5). Indeed, this estimate can be derived parallel to the smoothing estimates in Proposition 4.4. Therefore, using the properties of the diffeomorphism $\Phi _{\tilde{V}}$ again, we observe that

6.18 $$\begin{aligned} \Vert \tilde{g}_\tau \Vert _{L^{\infty }} \lesssim \Vert \tilde{g}_\tau \Vert _{\tilde{V}} = \Vert \Phi _{\tilde{V}}(\tilde{h}_c(\tau ))\Vert _{\tilde{V}} \lesssim \varepsilon \tau .\end{aligned}$$

Let us summarize what we have obtained so far. We showed there is small $\delta _0>0$ and $c_0>0$ such that if $\varepsilon _0 \tau <c_0$ and $\varepsilon <\varepsilon _0$, then there is a stationary solution $\tilde{V}(1+\tilde{g}_\tau )$ with the estimates (6.14), (6.16), (6.17), and (6.18). We shall now transform $\tilde{g}_\tau$ into the solution $\hat{g}_\tau$ of (3.7) that we are looking for. We thus set

6.19 $$\begin{aligned} \hat{g}_\tau = (g_0+1)\tilde{g}_\tau + g_0.\end{aligned}$$

Recalling that *h*(*t*) and $\tilde{h}(t)$ are related by the same transformation, $h(t) = (g_0+1)\tilde{h}(t)+g_0$, and using the estimates (6.5), we have that

$$\begin{aligned} \Vert h(t) - \hat{g}_r\Vert _V = \Vert (g_s+1)(\tilde{h}(t) - \tilde{g}_r)\Vert _V \lesssim \Vert \tilde{h}(t) - \tilde{g}_r\Vert _{\tilde{V}}. \end{aligned}$$

It remains to use the estimates in (6.14) and (6.17) together with the triangle inequality to deduce

$$\begin{aligned} \sup _{t\in [\tau ,\tau +2]}\Vert h(t) - \hat{g}_\tau \Vert _V \lesssim (\varepsilon \tau )^2 + \varepsilon \tau e^{-\frac{1}{8}\tilde{\lambda }\tau } + o(\varepsilon \tau ).\end{aligned}$$

First choose $\tau$ large (independently from $\tilde{V}$ thanks to (6.15)) and then $\varepsilon _0$ sufficiently small to satisfy $\varepsilon _0\tau <c_0$ and (6.7)

$$\begin{aligned}\sup _{t\in [\tau ,\tau +2]} \Vert h(t)-\hat{g}_\tau \Vert _V \leqq \frac{1}{2} \varepsilon \end{aligned}$$

for any $$\varepsilon \le \varepsilon _0$$.

Finally, (6.8) is an immediate consequence of (6.18) and the definition of $\hat{g}_\tau$ in (6.19).

We now have all the tools at hand to proceed to the proof of the exponential convergence result.

### Proof of Theorem 6.1

The idea is to iterate Proposition 6.6. By a translation in time, we may assume that

$$\begin{aligned} \sup _{t\geqq 0}\Vert h(t) \Vert _{L^\infty }\leqq \varepsilon _1 \end{aligned}$$

for some $\varepsilon _1 \leqq \min (\delta _0,\varepsilon _0)$. Moreover, we choose $\varepsilon _1$ small so that $2C\varepsilon _1 <\delta _0$ holds, where $C<\infty$ is the constant in Proposition 6.6.

To start with, we consider the trivial solution family $g_{0,s}:=0$ for $s\geqq 0$, which trivially satisfies the hypothesis of Proposition 6.6 with $\varepsilon =\varepsilon _1$. Therefore there exists a family of stationary errors $\{\hat{g}_{0,s}\}_{s\geqq \tau }$ satisfying

$$\begin{aligned} \sup _{s\geqq \tau }\Vert \hat{g}_{0,s}\Vert _{L^\infty } \leqq C \varepsilon _1 \end{aligned}$$

and

$$\begin{aligned} \sup _{t\in [s,s+2]} \Vert h(t)- \hat{g}_{0,s} \Vert _{L^2_{p+1} }\leqq \frac{\varepsilon _1}{2} \end{aligned}$$

for all $s\geqq \tau$. Hence, the translated family $\{g_{1,s}\}_{s\geqq 0}$ where $g_{1,s} = \hat{g}_{0,s+\tau }$ satisfies again the hypothesis of Proposition 6.6, this time with $\varepsilon = \varepsilon _1/2$ and *h*(*t*) replaced by $h(t+\tau )$. We perform a series of iterations, leading to families $\{g_{k,s}\}_{s\geqq 0}$ for any $k\in \textbf{N}$ satisfying

6.20 $$\begin{aligned} \sup _{t\in [s,s+2]} \Vert h(t+k\tau )- g_{k,s} \Vert _{L^2_{p+1} }\ \leqq \frac{\varepsilon _1}{2^k} \end{aligned}$$

and

6.21 $$\begin{aligned} \Vert g_{k-1,s}- g_{k,s }\Vert _{L^\infty } \leqq \frac{C\varepsilon _1}{2^{k-1}} \end{aligned}$$

for all $s\geqq 0$. Notice that the latter and the fact that we started with the trivial solution $g_{0,s} = 0$ entails that

$$\begin{aligned}\Vert g_{k,s} \Vert _{L^\infty } \leqq \sum _{\ell =1}^k \Vert g_{k-\ell +1,s} - g_{k-\ell ,s} \Vert _{L^\infty }\leqq 2C\varepsilon _1 \sum _{\ell =1}^k 2^{-k} \leqq 2C\varepsilon _1< \delta _0,\end{aligned}$$

by our choice of $\varepsilon _1$, which guarantees that condition (6.5) holds true in every iteration step.

Moreover, the estimate (6.21) also implies that, for any fixed *s*, the sequence $g_{k, s}$ converges geometrically in $L^\infty$ to some $g_{\infty , s}$,

$$\begin{aligned} \Vert g_{k,s} - g_{\infty ,s}\Vert _{L^{\infty }} \leqq \frac{C\varepsilon _1}{2^k}, \end{aligned}$$

for any $k\in \textbf{N}$ and $s\geqq 0$. We conclude via the triangle inequality and estimate (6.20) that

$$\begin{aligned} \Vert h(t+k\tau ) - g_{\infty ,s}\Vert _{L^2_{p+1} }\ \leqq C_1 2^{-k} \end{aligned}$$

for some new constant $C_1$, any $s\geqq 0$ and any $t\in [s,s+2]$. Picking $s=t=0$, we deduce exponential convergence with rate $\gamma = (\log 2)/\tau$ towards $g_{\infty ,0}$, which must actually vanish, $g_{\infty ,0}=0$, because *h*(*t*) is decaying to zero by the virtue of the Bonforte–Grillo–Vázquez theorem [12], cf. (1.5). This finishes the proof of the second dichotomy.

## Data Availability

Data sharing is not applicable to this article as no data sets were generated or analyzed during the current study.
